# Systematic evaluation of AML-associated antigens identifies anti-U5 SNRNP200 therapeutic antibodies for the treatment of acute myeloid leukemia

**DOI:** 10.1038/s43018-023-00656-2

**Published:** 2023-10-23

**Authors:** Katherine Knorr, Jahan Rahman, Caroline Erickson, Eric Wang, Mara Monetti, Zhuoning Li, Juliana Ortiz-Pacheco, Andrew Jones, Sydney X. Lu, Robert F. Stanley, Maria Baez, Nina Fox, Cynthia Castro, Alessandra E. Marino, Caroline Jiang, Alex Penson, Simon J. Hogg, Xiaoli Mi, Hideaki Nakajima, Hiroyoshi Kunimoto, Koutarou Nishimura, Daichi Inoue, Benjamin Greenbaum, David Knorr, Jeffrey Ravetch, Omar Abdel-Wahab

**Affiliations:** 1 Molecular Pharmacology Program, Sloan Kettering Institute, Memorial Sloan Kettering Cancer Center, New York, NY USA; 2 Laboratory of Molecular Genetics and Immunology, Rockefeller University, New York, NY USA; 3 Center for Hematologic Malignancies, Memorial Sloan Kettering Cancer Center, New York, NY USA; 4 The Jackson Laboratory for Genomic Medicine, Farmington, CT USA; 5 Stanford University School of Medicine, Stanford, CA USA; 6 Department of Stem Cell and Immune Regulation, Graduate School of Medicine, Yokohama City University, Yokohama, Japan; 7 Department of Hematology–Oncology, Institute of Biomedical Research and Innovation, Foundation for Biomedical Research and Innovation at Kobe, Kobe, Japan; 8 Computational Oncology, Department of Epidemiology and Biostatistics, Memorial Sloan Kettering Cancer Center, New York, NY USA; 9 Physiology, Biophysics & Systems Biology, Weill Cornell Medicine, Weill Cornell Medical College, New York, NY USA

**Keywords:** Acute myeloid leukaemia, Applied immunology, Immunotherapy, Cancer, Cancer therapy

## Abstract

Despite recent advances in the treatment of acute myeloid leukemia (AML), there has been limited success in targeting surface antigens in AML, in part due to shared expression across malignant and normal cells. Here, high-density immunophenotyping of AML coupled with proteogenomics identified unique expression of a variety of antigens, including the RNA helicase U5 snRNP200, on the surface of AML cells but not on normal hematopoietic precursors and skewed Fc receptor distribution in the AML immune microenvironment. Cell membrane localization of U5 snRNP200 was linked to surface expression of the Fcγ receptor IIIA (FcγIIIA, also known as CD32A) and correlated with expression of interferon-regulated immune response genes. Anti-U5 snRNP200 antibodies engaging activating Fcγ receptors were efficacious across immunocompetent AML models and were augmented by combination with azacitidine. These data provide a roadmap of AML-associated antigens with Fc receptor distribution in AML and highlight the potential for targeting the AML cell surface using Fc-optimized therapeutics.

## Abstract (web summary)

Abdel-Wahab and colleagues perform immunophenotyping of AML cells to identify AML-specific surface antigens and show that the RNA helicase U5 snRNP200 represents a therapeutic target for antibody therapy with optimized Fc receptor binding.

## Main

Following nearly 5 decades with few approved therapies for AML, the past 5 years have brought stellar progress, with the US Food and Drug Administration (FDA) approving several new therapies for patients with AML^1,2^. Despite these advances, 5-year survival for most adult patients with AML is less than 10%, illustrating the need for improved therapeutic approaches. While immunotherapies have revolutionized the treatment of many cancers, to date there are no effective immunotherapeutic agents for most patients with AML. One major challenge in developing antibody-based immunotherapies for AML, including therapeutic antibodies, antibody–drug conjugates and chimeric antigen receptor (CAR) T cells, has been identifying target antigens that effectively discriminate malignant cells from normal primitive hematopoietic stem and progenitor cells (HSPCs). This problematic ‘on-target off-tumor’ effect is illustrated by toxicities in patients with AML treated with therapies targeting CD33 and CD123 (refs. ^3–6^).

Most efforts to design antibody-based therapeutic approaches for AML have focused on selection of targets optimized for binding to Fab domains of antibodies. By contrast, optimization of therapeutic antibodies for AML through engineering the Fc region that engages with Fcγ receptors (FcγRs) on immune effector cells to elicit innate and adaptive anti-tumor responses has not been extensively explored. Modification of the antibody Fc region can guide preferential binding to FcγRs to activate signaling on immune effector cells, including induction of potent anti-tumor activity via antibody-dependent cellular cytotoxicity (ADCC) or antibody-dependent cellular phagocytosis as well as induction of cytotoxic CD8^+^ T cells following dendritic cell activation^7,8^. So far, over a dozen Fc-modified antibodies for enhanced FcγR binding have been approved by the FDA^7,8^. For example, strategies to enhance therapeutic efficacy through Fc-engineering modifications that increase binding to the activating CD16 receptor for the anti-CD20 antibody obinutuzumab^7^ and the anti-HER2 antibody margetuximab^9^ have been successful in improving responses in lymphoid and epithelial malignancies, respectively. However, the complexity of the FcγR system, with both activating and inhibitory receptors differentially expressed on discrete immune subsets, requires mapping FcγR abundance on immune effector cells within the tumor microenvironment.

Here, we provide a precise protein-level roadmap of AML-associated antigens as well as FcγR expression on immune cell subsets within the AML bone marrow microenvironment. In so doing, we describe a therapeutic antibody targeting an AML-associated antigen, U5 snRNP200, which we rigorously demonstrate is limited to malignant cells and not expressed on normal HSPCs. We demonstrate that the therapeutic activity of AML-targeting antibodies can be optimized by engineering to preferentially bind activating FcγRs and minimize interaction with inhibitory FcγRs. Finally, we identify that a standard-of-care agent in AML therapy, azacitidine, can favorably alter FcγR expression, yielding an improved ratio of activating to inhibitory receptor expression.

Collectively, these results have the potential to guide and redirect the design of antibody-based therapies for AML through optimization of both the antibody Fab and Fc regions to specifically target AML cells, limit off-target hematologic toxicity and maximize expression and engagement of activating FcγRs on immune effector cells within the bone marrow.

## Results

### U5 snRNP200 expression on AML versus normal cells

**Fig. 1:**
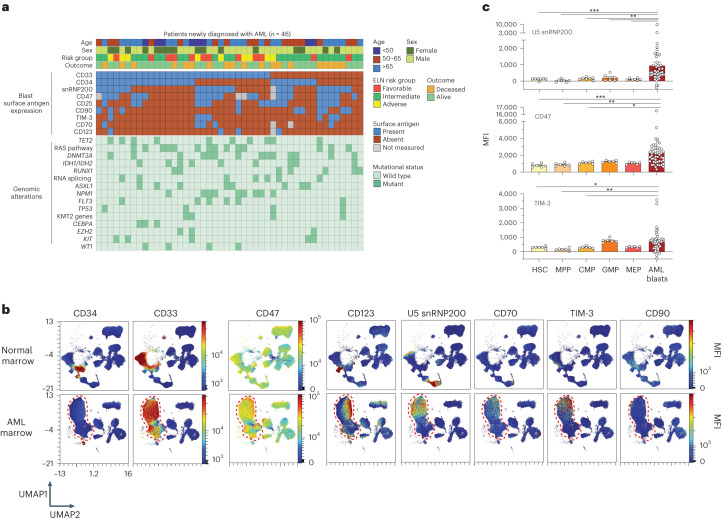
High-density immunophenotyping of AML surface antigen expression identifies AML-associated antigens. **a**, Oncoprint summarizing AML patient characteristics, clinical parameters and expression of AML-associated antigens on bone marrow leukemic blasts. ELN, European LeukemiaNet. **b**, Representative UMAPs comparing control and age-matched AML patient bone marrow samples subjected to 36-parameter phenotyping. Heatmap colors indicate relative surface antigen expression intensity. Red dashed lines indicate unbiased identification of malignant blasts. **c**, Median fluorescent intensity (MFI) of surface antigen expression on normal bone marrow HSPCs (*n* = 7 donors) versus AML blasts across patients (*n* = 46). HSCs (Lin^−^CD34^+^CD45^dim^CD90^+^CD38^−^); MPP (Lin^−^CD34^+^CD45^dim^CD90^−^CD38^−^); CMP, common myeloid progenitor (Lin^−^CD34^+^CD45RA^−^CD38^+^CD123^+^); GMP, granulocyte–macrophage progenitor (Lin^−^CD34^+^CD45RA^+^CD38^+^CD123^+^); MEP, megakaryocyte–erythroid progenitor (Lin^−^CD34^+^CD45RA^−^CD38^+^CD123^−^); *P* values are from the Mann–Whitney test: U5 snRNP200 blasts versus HSCs, ***P = 0.0006; blasts versus MPPs, ***P = 0.0001; blasts versus CMPs, **P = 0.0016; blasts versus granulocyte–macrophage progenitors, **P = 0.0068; blasts versus MEPs, ***P = 0.0004; CD47^+^ blasts versus HSCs, ***P = 0.0005; blasts versus MPPs, **P = 0.0022; blasts versus CMPs, *P = 0.0202; blasts versus MEPs, *P = 0.0101; TIM-3^+^ blasts versus HSCs, *P = 0.0275; blasts versus MPPs, **P = 0.0028; blasts versus CMPs, *P = 0.0326. Data are mean ± s.e.m. Source data

We developed a custom 36-parameter spectral flow cytometry panel optimized to simultaneously interrogate AML blast surface phenotype, normal HSPC subsets, mature immune cells and individual activating and inhibitory FcγRs in human bone marrow (Supplementary Table 1). This assay included profiling of antigens (CD123, TIM-3, CD33, CD47, CD90, CD38, CD25, CD70 and U5 snRNP200) being actively assessed in clinical trials^10–12^ or previously described as putative AML-associated antigens^13–15^. U5 snRNP200 was specifically included based on prior identification of cell surface U5 snRNP200 protein expression on AML cells^15^. In this prior study, antibodies directed against U5 snRNP200 were identified as produced in donor B cells from patients with AML in long-term remission after allogeneic HSC transplantation, suggesting that anti-U5 snRNP200 antibodies may be responsible for effective graft-versus-leukemia effect. Our antibody panel was applied to bone marrow samples from 46 newly diagnosed clinically and genetically annotated adult patients with AML (Supplementary Table 2). This cohort represents the heterogeneous features of newly diagnosed patients with AML with a median age of 58 years and with 65% of patients being of adverse risk, respectively, according to 2022 European LeukemiaNet risk classification^1^ (Fig. 1a and Supplementary Table 2). The median follow-up for the cohort is 4.3 years.

**Fig. 2:**
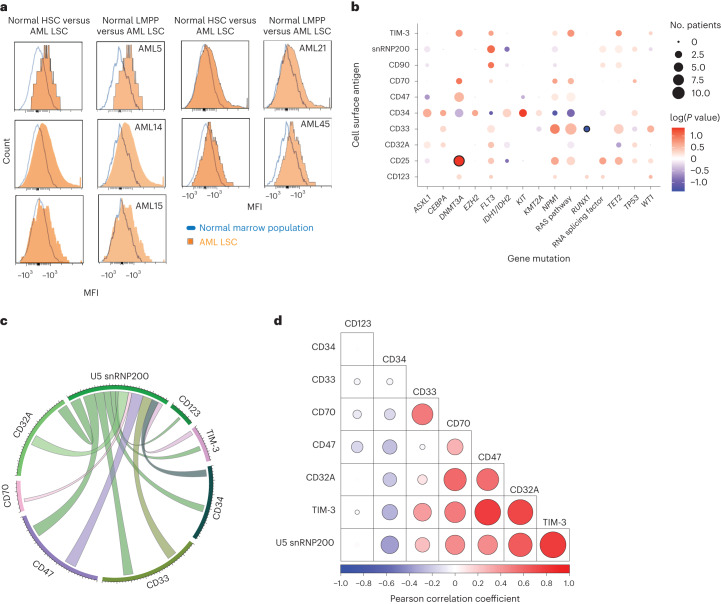
Cell surface expression of U5 snRNP200 in AML and coexpression patterns with other AML-associated antigens. **a**, Histograms of MFI on live HSCs (live Lin^−^CD34^+^CD45^dim^CD90^+^CD38^−^) and lympho-primed MPP cells (LMPPs; live Lin^−^CD34^+^CD38^−^CD90^−^CD45RA^+^ cells) from normal bone marrow (blue lines) versus on AML leukemia stem cells (LSCs; live CD34^+^CD38^−^CD90^−^CD45RA^+^ cells) for five distinct patients with AML whose bulk leukemic cells express cell surface U5 snRNP200. **b**, Bubble plot summarizing surface antigen expression and mutation status patterns. Bubbles with dark circle outlines highlight patterns that reach statistical significance (that is, CD25 expression and *DNMT3A* mutations co-occur, whereas CD33 and *RUNX1* mutations are mutually exclusive). log-transformed *P* values were determined by one-sided Fisher test, with positive values indicating positive associations and negative values indicating negative associations. **c**, Circos plot depicting coexpression between U5 snRNP200 and known AML-associated surface antigens on AML patient bone marrow blasts. **d**, Correlogram depicting Pearson correlation coefficients of surface antigen intensity on AML patient bone marrow blasts. Source data

**Extended Data Fig. 1:**
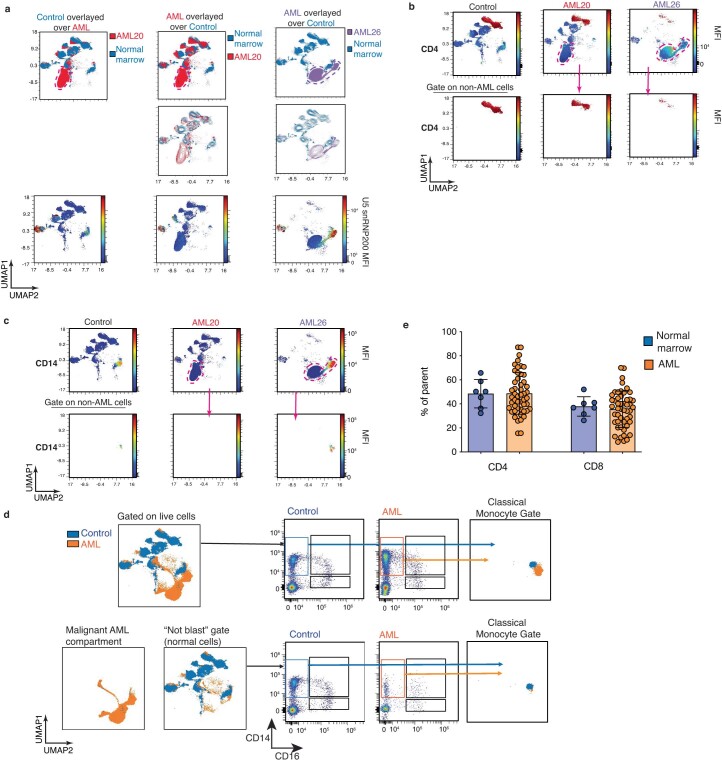
High density immunophenotyping UMAP gating strategies eliminate blast contamination in normal cell populations and summary of antigen/mutation co-expression patterns. **(a)** UMAP overlay examples showing normal cell populations as defined in normal donor sample. Malignant AML cell populations (in red and purple dotted outlines) form individual cell islands that do not overlap with any normal populations. Top left panel: normal donor sample cell islands (in blue) overlayed on AML sample 20 (in red). Top middle panel: same normal donor and AML sample 20 as depicted in top left panel but here the AML sample 20 is overlayed on the normal donor sample. Top right panel: AML sample 26 (purple) overlayed on the same normal donor sample (blue). The panels in the middle rows are identical UMAPs depicted in top rows but displayed in contour form rather than solid color form. The panels in the third row are identical UMAPs depicted in top rows but contain a heatmap indicate relative surface antigen expression intensity of U5 snRNP200. **(b)** UMAP examples demonstrating preservation of normal cell islands between normal donor and AML samples. Colorimetric overlay is CD4 in top and bottom panels (that is CD4 T cell island is red). To eliminate malignant AML cell contamination (‘blasts’) while analyzing normal cell populations through manual gating, the malignant AML blast cell island was gated in the top panel UMAP and excluded from subsequent manual gating analysis (outside pink dotted line, ‘not gate’). Bottom panels depict UMAPs filtered to display resulting manually gated CD4 T cells and no changes are seen in these populations compared with original ungated UMAP (top panel). **(c)** Same UMAP examples as featured in (b) but with CD14 colorimetric overlay. Again, blasts were excluded through gating on the UMAP (pink dotted line) and subsequently manual gating (outside pink dotted line, ‘not gate’) was performed to identify normal cell populations without blast contamination. Bottom panels depict UMAPs filtered to display manually gated immature monocytes (present in control and one of two AML samples) and no changes are seen in these cell islands compared with original ungated UMAP (top panel). **(d)** Example of UMAP gating strategy for exclusion of malignant AML cells via UMAP ‘blast gate’ prior to quantification and analysis of immune cell subsets by traditional manual gating. Top left UMAP depicts all live cells from one normal donor (blue) and one AML sample (orange); bottom left UMAP depicts malignant AML cells isolated by the ‘blast’ gate (that is cell island unique to AML sample) and bottom right UMAP depicts all cells outside of the ‘blast’ gate (that is the ‘not blast’ gate). Middle panels contain scatter plots depicting manual gating of classical monocytes in normal donor (blue gate) and AML sample (orange gate) comparing results when gated from all live cells (top row) versus ‘not blast’ gate (bottom row). Right panels depict subsequent UMAP images generated from classical monocyte gates from normal donor (blue) and AML sample (orange) either from the all live cell gating approach (top) or the approach where blasts are excluded from analysis (bottom). **(e)** Frequency of CD4 and CD8^+^ T cells in AML bone marrow (orange, n = 50 patients) compared to normal donor bone marrow (blue, n = 7 donors). Bar graphs represent the mean ± SEM. Source data

Using live cell populations from bone marrow samples from six unaffected donors (median age, 41.5 years) and patients with AML as input, we generated uniform manifold approximation and projections (UMAPs) to objectively delineate the malignant blast compartment from normal cell populations in an unbiased manner (Fig. 1b and Extended Data Fig. 1a). One of the main challenges of current AML antibody-based therapeutics is on-target off-tumor side effects due to expression of the antibody target on normal HSPCs^10,16^. Comparison of surface expression of antigens under evaluation for AML therapeutic targeting revealed increased abundance of CD47 (*P* = 0.0005), TIM-3 (*P* = 0.028) and U5 snRNP200 (*P* = 0.0006) on the surface of AML cells relative to normal hematopoietic stem cells (HSCs) (Lin^−^CD34^+^CD45^dim^CD90^+^CD38^−^) from age-matched healthy individuals, consistent with prior reports^15,17,18^ (Fig. 1c). At the same time, the most significant differentially expressed antigen between AML blasts and normal CD34^+^ hematopoietic precursors was U5 snRNP200, as this antigen (originally identified as a potential AML-specific antigen in prior work^15^) was totally absent from normal HSCs, multipotent progenitors (MPPs) and any downstream myeloid progenitor population (Fig. 1c). Of note, U5 snRNP200 was present on blasts from 50% of newly diagnosed patients with AML. In patients with AML in whom U5 snRNP200 cell surface expression was detected on bulk CD34^+^ malignant cells, U5 snRNP200 was also present on immunophenotypically defined leukemia stem cells (Fig. 2a).

We next examined the co-occurrence of mutations with antigen expression on blasts in this cohort of newly diagnosed adult patients with AML. This revealed a statistically significant positive association between *DNMT3A* mutations and surface CD25 expression as well as a significant negative association in which patients with mutations in *RUNX1* tended to have less CD33 on AML blasts (Fig. 2b). The association between CD33-positive blasts and *NPM1* mutation was captured in our data despite not reaching statistical significance^19,20^. Importantly, however, there were no clear statistically significant associations between any genetic alterations and surface U5 snRNP200 expression in this cohort. Moreover, there was no statistically significant association between U5 snRNP200 cell surface expression and age at diagnosis, AML risk group, sex or outcome at time of analysis (living or deceased).

Coexpression of antigens on AML blasts was also analyzed with the aim of defining antigen combinations suitable for multispecific or bispecific antibodies or multi-antigen CAR T cell therapy, approaches being actively pursued in hopes of reducing on-target off-tumor side effects. Indeed, we observed patterns of antigen coexpression on AML blasts (Fig. 2c,d) including statistically significant coexpression of U5 snRNP200 with CD47 (*P* = 0.002) and TIM-3 (*P* < 0.0001), two antigens under evaluation using separate therapeutic antibodies in phase 2–3 clinical trials for patients with AML or myelodysplastic syndrome currently^21^.

### Skewed Fc receptor distribution in the AML microenvironment

**Fig. 3:**
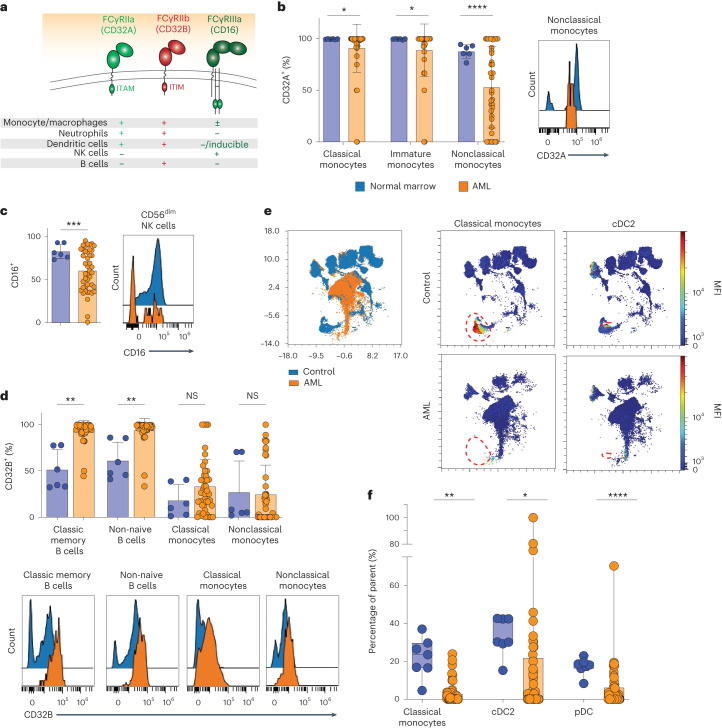
Alterations in frequencies and distribution of Fc receptor expression on immune cell subsets in the bone marrow microenvironment of patients with AML. **a**, Schematic summary of FcγR expression across normal immune cell subsets. ITAM, immunoreceptor tyrosine-based activating motif; ITIM, immunoreceptor tyrosine-based inhibitory motif. **b**, Expression of activating receptor CD32A on monocyte populations in AML bone marrow (orange, *n* = 44 patients) compared to bone marrow from unaffected donors (blue, left, *n* = 6 donors) and representative flow cytometry histogram of CD32A expression on AML nonclassical monocytes (orange) compared to controls from unaffected donors (blue, right); *P* values are from Welch’s unpaired *t*-test. *P = 0.0182 (classical monocytes), *P = 0.0193 (immature monocytes), ****P < 0.0001 (left). **c**, Expression of activating receptor CD16 on CD56^dim^ NK cells in AML bone marrow (*n* = 44 patients) compared to bone marrow from unaffected donors (left, *n* = 6 donors) and a representative flow cytometry histogram (right); *P* values are from Welch’s unpaired *t*-test. ***P = 0.0002 (left). **d**, Inhibitory receptor CD32B on B cells and monocytes in AML bone marrow (*n* = 44 patients) compared to bone marrow from unaffected donors (*n* = 6 donors) (top) and representative flow cytometry histograms (bottom); *P* values are from Welch’s unpaired *t*-test. **P = 0.0048 (classic memory B cells) and 0.0094 (non-naive B cells) (top). NS, not significant. **e**, Representative UMAP overlay generated from the 36-color spectral flow cytometry panel comparing normal bone marrow (blue) and AML bone marrow (orange, left) and individual representative UMAPs depicting classical monocyte and cDC2 cell populations (each demarcated with a red outline) in an unaffected control donor (top) compared to a patient with AML (bone marrow) in whom these populations are absent (bottom). **f**, Quantification of classical monocytes and cDC2 cells in AML bone marrow (*n* = 49) compared to bone marrow from control donors (*n* = 7 donors); *P* values are from Welch’s unpaired *t*-test. **P = 0.0028 (classical monocytes), **P = 0.0014 (cDC2) and ****P < 0.0001 (pDC). Data are mean ± s.e.m. Source data

There is abundant evidence that tumor-targeting antibodies with Fc regions optimized to activate immune cell subsets have greater anti-tumor effects than antibodies that do not engage immune cell subsets^7^. However, the precise distribution of Fc receptors on immune cell subsets present in the AML bone marrow microenvironment has not previously been explored. To address this, we integrated antibodies specific for the activating receptors CD32A (also known as FcγRIIA) and CD16 (FcγRIIIA) as well as the inhibitory receptor CD32B (FcγRIIB) (Fig. 3a) into our custom 36-parameter flow cytometry panel. This panel captures all Fc receptor-expressing immune effector cells that contribute to ADCC (classical, immature and nonclassical monocytes, natural killer (NK) cells) and other immune cell populations that express Fc receptors (conventional, plasmacytoid and monocyte-derived dendritic cells as well as B cells, plasmablasts and basophils). Importantly, the strategy of generating UMAP projections overlaying samples from unaffected donors and patients with AML allows for unbiased demarcation of the malignant cell population, which can be readily identified on the UMAP projection and eliminated from immune cell analysis (Extended Data Fig. 1b,c). Exclusion of malignant AML cells from normal cell populations is essential, given the potential overlap of expression of immune cell markers on leukemic cells, which can compromise the integrity of manual gating strategies^22^ (Extended Data Fig. 1d).

Comparison of expression of the activating Fc receptors CD32A and CD16 on mature immune cells in bone marrow of unaffected individuals versus those with AML revealed significant downregulation of the activating receptor CD32A on classical (CD14^+^CD16^−^; *P* = 0.02), immature (CD14^+^CD16^+^; *P* = 0.02) and nonclassical (CD14^−^CD16^+^; *P* < 0.0001) monocytes (Fig. 3b) as well as downregulation of activating receptor CD16 on CD56^dim^ NK cells (*P* = 0.0002) in patients with AML (Fig. 3c). Furthermore, there was increased expression of the inhibitory Fc receptor CD32B on classic memory B cells (CD20^+^CD19^+^IgD^−^; *P* = 0.005) and non-naive B cells (CD20^+^CD19^+^IgD^−^ and CD20^+^CD19^+^IgD^+^CD27^+^; *P* = 0.009) as well as classical and nonclassical monocytes in AML marrow compared to those from healthy control individuals (Fig. 3d). Moreover, patients with AML had a significantly lower frequency of classical monocytes (*P* = 0.003), type 2 conventional dendritic cells (cDC2; *P* = 0.001) and plasmacytoid dendritic cells (*P* < 0.0001), cell types required for ADCC and antigen presentation, respectively, in their marrow than unaffected individuals (Fig. 3e,f). Finally, there was no significant difference in the frequency of T cell populations in the bone marrow of newly diagnosed patients with AML and unaffected donors, consistent with prior reports^23^ (Extended Data Fig. 1e).

Overall, these data identify a previously unrecognized imbalance in the ratio of activating to inhibitory Fc receptors in the immune microenvironment of AML. In particular, the adult AML bone marrow is characterized by a greater proportion of immune effector cells expressing inhibitory Fc receptors as well as fewer classical monocytes, cDC2 cells and plasmacytoid dendritic (pDC) cells than in unaffected individuals.

### Surface membrane regulation of U5 snRNP200 in AML

U5 snRNP200 is an ATP-dependent RNA helicase 250 kDa in size, which is an essential, evolutionarily conserved core component of the spliceosome^24^. Its function and molecular mechanism have been exquisitely defined as serving to unwind duplex RNA formed by U4 and U6 small nuclear RNA required for formation of the catalytic spliceosome^25^. It was therefore unexpected that a nuclear enzyme involved in RNA splicing would be present on the cell membrane^15^.

**Fig. 4:**
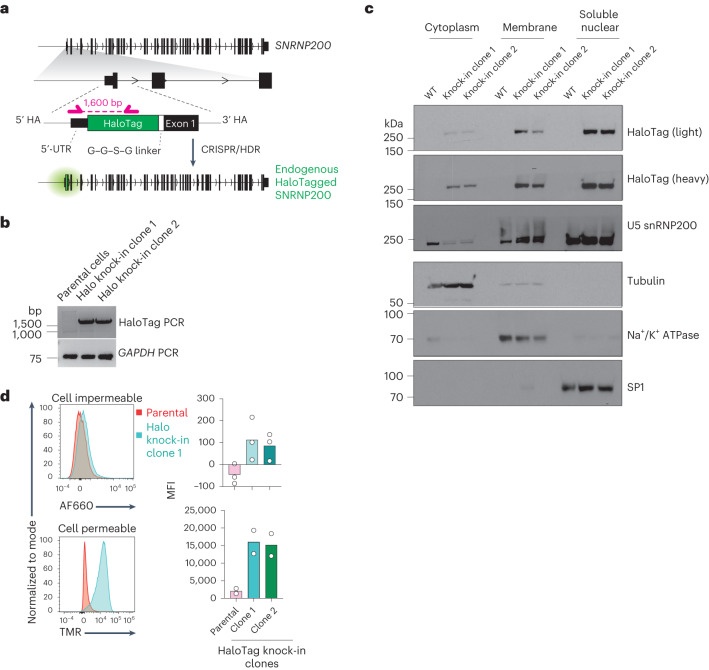
Genetic validation of cell surface membrane U5 snRNP200 expression and determinants of U5 snRNP200 cell surface membrane localization. **a**, Schema of the vector engineered for knock-in of the sequence for N-terminal HaloTag into the *SNRNP200* locus in the human AML cell line K562. UTR, untranslated region. HA, homology arm. **b**, PCR amplification of the HaloTag sequence for verification of expression in HaloTag–U5 snRNP200-expressing K526 cells. Representative of three independent experiments. **c**, Western blot for HaloTag (two exposure times are shown and denoted as light and heavy) and U5 snRNP200 in subcellular fractions of K562 cell clones expressing HaloTagged endogenous U5 snRNP200. Loading controls for cell fractions include tubulin (cytoplasmic), Na^+^/K^+^ ATPase pump (membrane) and specificity protein 1 (SP1; soluble, nuclear). WT, wild type. **d**, Representative flow cytometry histograms of MFI values (left) for cell-impermeable (top) and cell-permeable (bottom) fluorescent HaloTag ligands in K562 cells from **b** and quantification of signal (right; mean value is shown and each dot represents a data point from an independent experiment). AF660, Alexa Fluor 660. TMR, tetramethylrhodamine. Source data

Given that antibody-based detection of U5 snRNP200 alone may not reliably prove the presence of full-length U5 snRNP200 on the plasma membrane, we sought to rigorously validate this observation by introducing the sequence encoding a HaloTag epitope in frame into the sequence for the N terminus of the protein encoded by *SNRNP200* in K562 human AML cells using CRISPR-mediated homology directed repair (HDR) editing (Fig. 4a,b). Subcellular fractionation of HaloTag knock-in K562 cell clones and controls followed by western blotting for HaloTag and U5 snRNP200 confirmed the presence of endogenous U5 snRNP200 in the nuclear fraction in parental K562 cells and at its full size of 250 kDa in the two HaloTag knock-in clones. Moreover, western blotting of lysates from distinct cellular compartments revealed localization of full-length U5 snRNP200 (as indicated by the HaloTag) on the cell membrane (Fig. 4c). We further confirmed localization of endogenous U5 snRNP200 at the cell membrane using cell-impermeable fluorescent ligands that interact with the HaloTag (Fig. 4d). At the same time, the abundance of cell membrane-localized U5 snRNP200 was only a fraction of U5 snRNP200 present within the cell, as revealed by membrane-permeable fluorescent ligands that interact with the HaloTag (Fig. 4d). These data indicate that the N terminus of U5 snRNP200 is extracellular on the surface of AML cells.

**Fig. 5:**
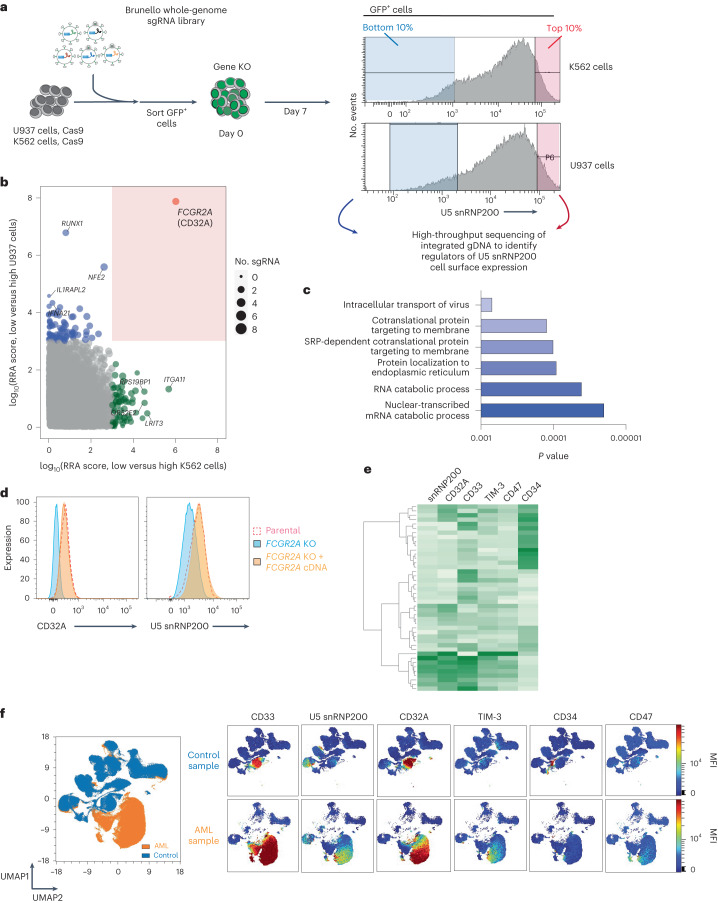
Determinants of U5 snRNP200 cell surface membrane localization on the AML cell surface and coexpression with CD32A. **a**, Schema of the whole-genome CRISPR screen to identify genes positively and negatively associated with U5 snRNP200 cell surface expression on AML cells. As shown, the Brunello sgRNA library (via GFP^+^ lentivirus) was stably introduced in K562 and U937 cells, and, subsequently, the top 10% and bottom 10% of U5 snRNP200-surface expressing GFP^+^ cells were sorted for sgRNA sequencing. **b**, Statistically significant sgRNA species associated with low U5 snRNP200 expression in U937 (*y* axis) and K562 (*x* axis) cells. RRA, robust rank aggregation. As shown, knockout of *FCGR2A* (which encodes FcγRIIA or CD32A) was significantly associated with low cell surface snRNP200 expression across both cell lines. **c**, GO analysis of genes required for cell surface U5 snRNP200 expression from the CRISPR screen in **e**. **d**, Histograms of CD32A (left) and U5 snRNP200 (right) expression on U937 cells following stable knockout of *FCGR2A* and re-expression of CD32A using cDNA impervious to knockout. Histogram flow cytometry plots are representative of five flow cytometry experiments performed using these cell lines. **e**, Heatmap depicting coexpression patterns of antigens on AML blasts including coexpression of U5 snRNP200 and CD32A. **f**, UMAP overlay comparing samples from unaffected donors and patients with AML for identification of malignant AML cells (left, isolated orange cell island) and colorimetric overlays of known and new AML antigens for antibody targeting (right) facilitate visualization of expression on AML cells versus normal cells as well as coexpression patterns on AML cells. SRP, signal recognition particle. Source data

Given the unexpected presence of surface membrane U5 snRNP200, we next sought to determine the molecular regulators of surface U5 snRNP200 expression. We applied the genome-wide Brunello single-guide RNA (sgRNA) library^26^ to two human AML cell lines expressing cell surface U5 snRNP200 (K562 and U937 cells) and sorted the highest (top 10%) and lowest (bottom 10%) surface U5 snRNP200-expressing populations (Fig. 5a). Sequencing of sgRNA species in these two populations revealed that knockout of *FCGR2A*, the gene that encodes the activating Fc receptor CD32A, was highly associated with loss of surface U5 snRNP200 expression in both cell lines (*P* < 0.05; Fig. 5b). Moreover, Gene Ontology (GO) analysis indicated numerous genes encoding proteins required for subcellular protein trafficking that were also required for cell surface U5 snRNP200 expression (Fig. 5c).

To confirm the role of CD32A in surface U5 snRNP200 expression, we performed flow cytometry using U937 cells with CRISPR-mediated stable knockout of *FCGR2A* using an sgRNA independent from those used in the CRISPR screen. Flow cytometric staining for CD32A confirmed diminished expression in *FCGR2A*-knockout cells (Fig. 5d). Importantly, U5 snRNP200 cell surface expression mirrored that of CD32A, as U5 snRNP200 cell surface abundance was also abolished with *FCGR2A* knockout. Moreover, restoration of surface CD32A expression using *FCGR2A* cDNA impervious to sgRNA knockout rescued both cell surface U5 snRNP200 and CD32A expression (Fig. 5d). Consistent with these cell line data, a tight association between CD32A and U5 snRNP200 protein abundance on the surface of AML cells was also clear in patient specimens (Fig. 5e,f).

**Fig. 6:**
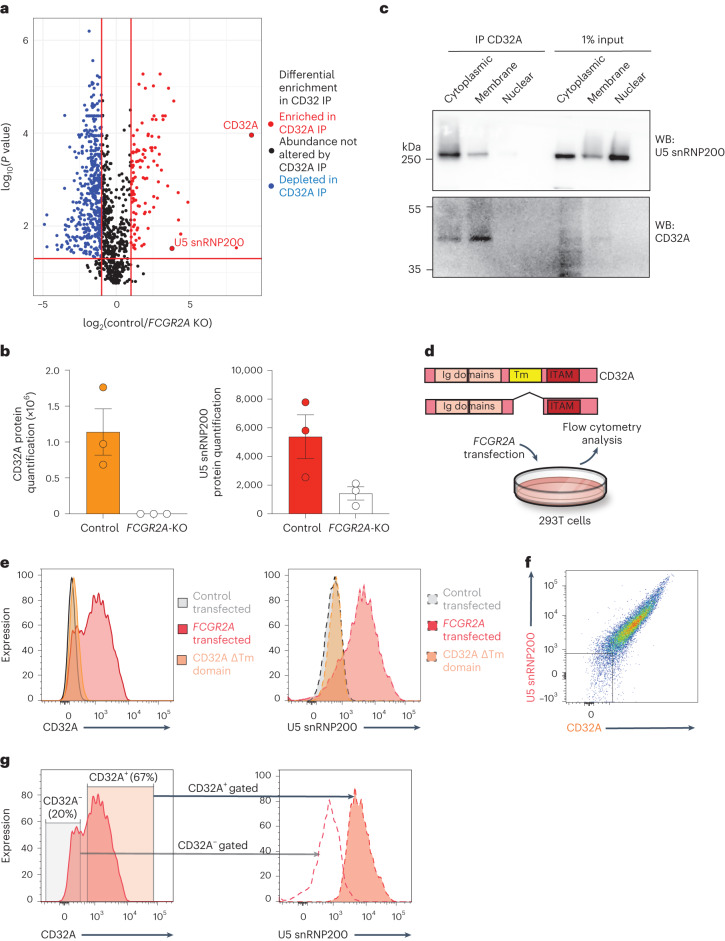
Physical interaction of CD32A and U5 snRNP200 at the AML cell membrane and requirement of the CD32A transmembrane domain for U5 snRNP200 surface membrane localization. **a**, Volcano plots of proteins differentially enriched in immunoprecipitation of CD32A from the membrane followed by mass spectrometry from wild-type versus knockout K562 cells. Proteins displayed were identified in *FCGR2A*-wild-type versus *FCGR2A*-knockout cells in triplicate, and values displayed are the mean of triplicate results. *P* values were derived by two-sided *t*-test, and *P* values were adjusted for multiple comparisons. **b**, Quantification of CD32A (left) and U5 snRNP200 (right) from immunoprecipitation–mass spectrometry of membrane-bound CD32A from *FCGR2A*-wild-type versus *FCGR2A*-knockout K562 cells. Each value represents data from a single mmunoprecipitation–mass spectrometry experiment with three biological replicates. **c**, Immunoprecipitation of CD32A followed by western blot in the cells from **a**. Representative of three independent experiments. **d**, Schematic of experiments to test the requirement of CD32A and its transmembrane (Tm) domain in the cell surface localization of U5 snRNP200 in 293T cells. **e**, Histograms of CD32A (left) and U5 snRNP200 (right) in 293T cells transfected with control, *FCGR2A*-wild-type cDNA or *FCGR2A* cDNA with in-frame deletion of the sequence for the transmembrane domain (‘CD32A ΔTm domain’). **f**, Representative flow cytometry plot of CD32A versus U5 snRNP200 surface expression in 293T cells transfected to express CD32A. **g**, Cell surface expression of U5 snRNP200 (right) in the cells from **d** gated on low versus high CD32A-expressing cells (left). Data are mean ± s.e.m. Source data

Given that CD32A is a known transmembrane protein, we hypothesized that the cell surface association with U5 snRNP200 occurs due to physical association of CD32A and U5 snRNP200 in AML cells. To test this hypothesis, we performed immunoprecipitation of CD32A in cell membrane protein fractions from control and *FCGR2A*-knockout K562 cells followed by mass spectrometry. This revealed a clear interaction of CD32A and U5 snRNP200 in the membrane of AML cells in K562 cells, and the specificity of this interaction was confirmed, as U5 snRNP200 was not detected in the cell membrane of *FCGR2A*-knockout K562 cells (Fig. 6a,b). Immunoprecipitation–mass spectrometry data were validated by immunoprecipitation–western blot experiments in which CD32A and U5 snRNP200 interacted in both the cell membrane and the cytoplasm. Finally, we identify that the cell membrane association of U5 snRNP200 is dependent on the transmembrane domain of CD32A (Fig. 6c). Transfection of 293T cells (which lack cell surface expression of CD32A or U5 snRNP200) to express CD32A resulted in cell surface localization of both proteins (Fig. 6d–g). In fact, the levels of CD32A cell surface abundance in the transfected cells was associated with cell surface U5 snRNP200 abundance. Conversely, cells transfected to express CD32A constructs that lack the transmembrane domain failed to express cell membrane U5 snRNP200 (Fig. 6e). These studies rigorously validate surface U5 snRNP200 expression on AML cells and elucidate CD32A as a key regulator of the physical association of U5 snRNP200 at the AML cell surface.

### Upregulation of viral RNA sensing in U5 snRNP200-high AML

**Fig. 7:**
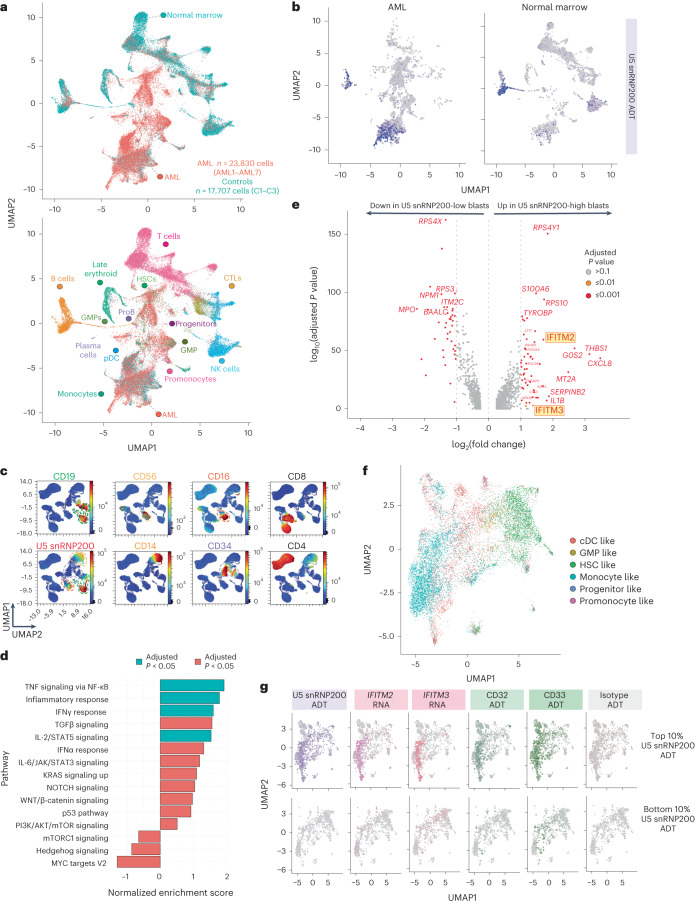
Unbiased evaluation of cell surface U5 snRNP200 expression in normal and malignant hematopoietic cells and transcriptional characteristics of cell surface U5 snRNP200-expressing AML cells. **a**, Multimodal UMAP projection delineating cell populations originating form normal and malignant AML bone marrow samples (top) and cell type labels (bottom). CTL, cytotoxic T lymphocytes; proB, pro-B cells. **b**, Representative U5 snRNP200 ADT colorimetric overlay on AML (left) and control donor (right) bone marrow cell populations. **c**, Representative UMAPs generated from the custom 36-parameter spectral flow cytometry panel displaying cell populations in control bone marrow. Heatmap colors indicate relative antigen expression intensity. Dashed lines indicate cell island subsets for comparison of surface U5 snRNP200 expression (bottom left): CD19^+^ B cells, green; CD56^+^ NK cell subset, yellow; CD16^+^ NK cell subset, red; CD14^+^ monocyte subset, orange; CD34^+^ HSCs, purple). **d**, Pathway enrichment observed in high U5 snRNP200-surface expressing AML cells. Enrichment score was calculated for a given gene set using log_2_-transformed fold change ranking when comparing U5 snRNP200-high versus U5 snRNP200-low populations and then normalized by the size of that gene set. To identify the *P* value, 1,000 random gene sets were generated, and an enrichment score was calculated for each of them. The *P* value was estimated as the number of random gene set enrichment scores with the same or more extreme values divided by the total number of randomly generated gene sets. For the adjusted *P* value, the Benjamini–Hochberg procedure was used. **e**, Differential gene expression in high versus low U5 snRNP200-surface expressing AML cells. *P* values were identified by two-sided implementation of the Wilcoxon rank-sum test. The *P* value was adjusted for multiple testing using Bonferroni correction. **f**, Dedicated AML cell UMAP depicts distinct proteogenomic subsets. **g**, Colorimetric overlay of surface expression of U5 snRNP200, CD33 and CD32 ADT signals along with *IFITM2* and *IFITM3* mRNA expression in the top 10% highest (top) and bottom 10% lowest or negative (bottom) U5 snRNP200-surface expressing AML cells. Source data

**Extended Data Fig. 2:**
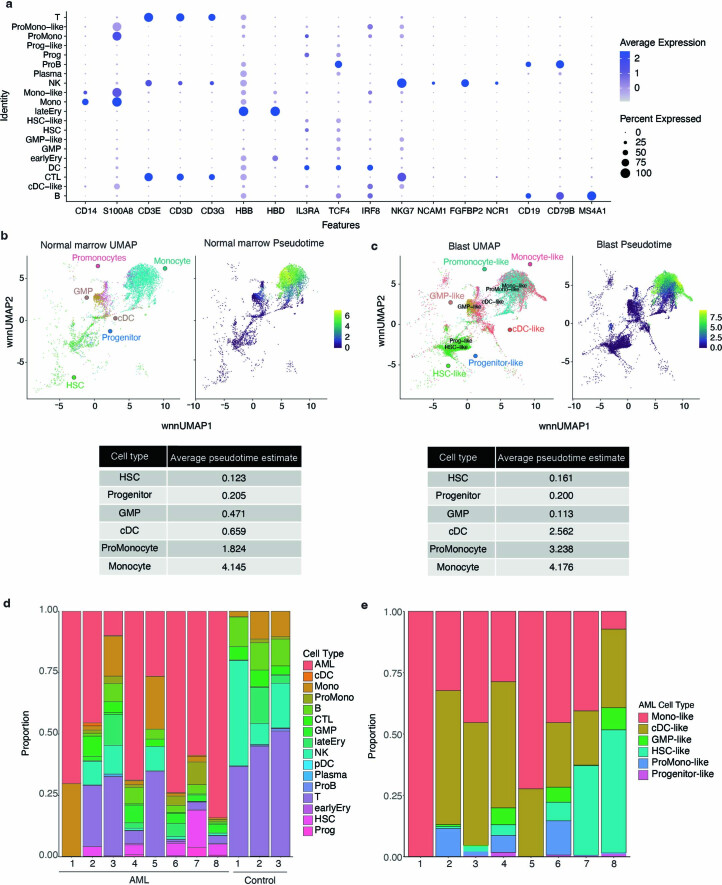
Validation of CITE-seq data. **(a)** Bubble plot demonstrating RNA expression levels of canonical markers within indicated cell cluster. **(b)** Normal donor and **(c)** AML blast pseudotime plots. **(d)** Normal donor and **(e)** AML cell type composition summaries. Source data

We next sought to evaluate the biological characteristics of AML blasts with upregulated cell surface U5 snRNP200. We employed cellular indexing of transcriptomes and epitopes by sequencing (CITE-seq)^27^ to simultaneously capture surface proteomes and gene expression. A panel of 131 oligonucleotide-tagged antibodies, including a custom antibody-derived tagged (ADT) anti-U5 snRNP200 antibody, was applied to 11 bone marrow samples including three from age-matched unaffected donors and eight newly diagnosed patients with AML (Supplementary Table 3). A total of 42,251 cells were mapped^28–30^ using gene expression signatures from a previously published annotated reference dataset of bone marrow samples from newly diagnosed patients with AML and healthy age-matched controls (Fig. 7a)^31^. Clusters were validated based on expression of lineage-specific gene expression and cell surface markers known to demarcate specific immune cell populations^32^ (Extended Data Fig. 2a) and exhibited reliable pseudotime estimates^33^ (Extended Data Fig. 2b,c), consistent with differentiative expectation. Samples from unaffected donors contained preserved normal immune cell subsets, whereas these populations were variable in patients with AML, consistent with previous reports^32,34^ (Extended Data Fig. 2d,e).

**Extended Data Fig. 3:**
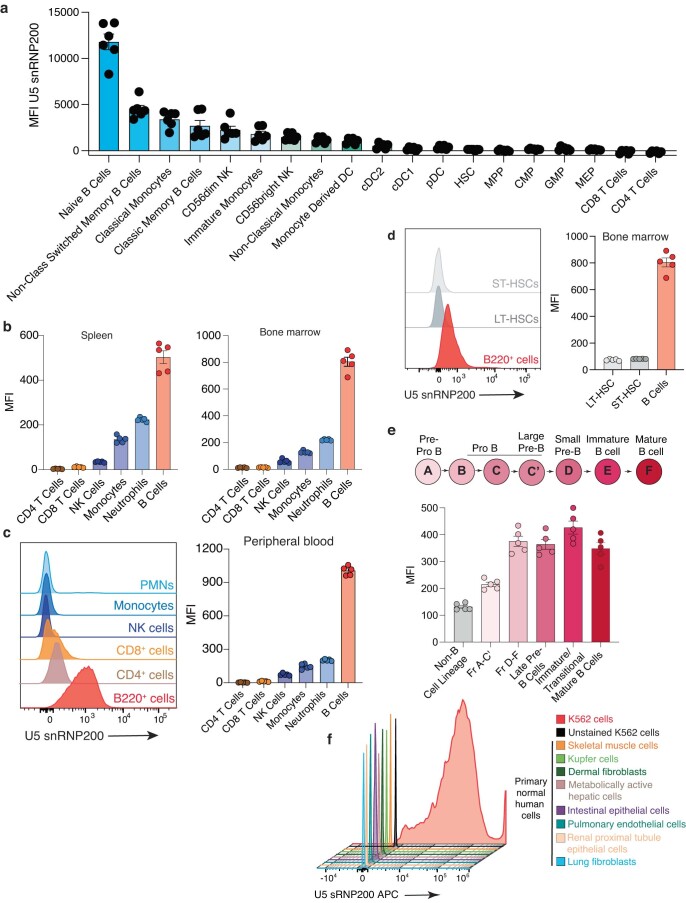
U5 snRNP200 expression in normal murine hematopoietic cells. **(a)** Median fluorescence intensity (MFI) values of U5 snRNP200 expression on immune cell populations from six normal adult bone marrow samples (n = 6 donors). Bar graphs represent mean ± SEM. **(b)** Quantification of U5 snRNP200 expression across immune cell subsets in the spleen and bone marrow (n = 5 mice). Bar graphs represent mean ± SEM. **(c)** Representative histogram (left panel) and quantification of U5 snRNP200 expression on peripheral blood immune cell subsets. n = 5 mice. Bar graphs represent mean ± SEM. **(d)** Representative histogram (left panel) and quantification of U5 snRNP200 expression on bone marrow stem cells compared to B-cells. n = 5 mice. Bar graphs represent mean ± SEM. **(e)** Quantification of U5 snRNP200 expression across B cell differentiation states. n = 5 mice. Bar graphs represent mean ± SEM. MFI = mean fluorescent intensity. **(f)** Histogram of U5 snRNP200 cell surface expression on K562 cells versus normal non-hematopoietic tissues stained for U5 snRNP200 versus unstained K562 cells. Source data

Projection of U5 snRNP200 ADT signals onto the UMAP cell clusters confirmed the absence of U5 snRNP200 surface expression on HSPCs while affirming the presence of cell surface U5 snRNP200 on AML cells (Fig. 7b). In addition, surface U5 snRNP200 was clearly present on B cells and a subset of NK cells (CD56^dim^ NK cells) and monocytes (classical monocytes) within bone marrow of unaffected donors and patients with AML (Fig. 7b). The expression pattern of cell surface U5 snRNP200 in unaffected donors was consistent across CITE-seq and spectral flow cytometry in which surface U5 snRNP200 was present on B cells (Fig. 7c, green outline), a subset of NK cells (Fig. 7c, yellow outline) and monocytes (Fig. 7c, orange outline) but not CD34^+^ cells (Fig. 7c, purple outline). The cell surface distribution of U5 snRNP200 on normal human immune cell populations from the bone marrow of six unaffected adult individuals is shown in Extended Data Fig. 3a. This expression pattern of U5 snRNP200 was conserved in mice, in which U5 snRNP200 was present across all bone marrow and spleen B cell subsets but absent on T cells and HSPC populations (Extended Data Fig. 3b–e). Evaluation of cell surface U5 snRNP200 expression on adult human tissues (including skeletal muscle cells, Kupffer cells, dermal fibroblasts, metabolically active hepatic cells, intestinal epithelial cells, pulmonary endothelial cells, renal proximal tubule epithelial cells and lung fibroblasts) revealed a clear absence of cell surface U5 snRNP200 (Extended Data Fig. 3f).

Following multimodal cell type identification of malignant populations by CITE-seq, AML cells were subsequently analyzed for differential gene expression profiles based on U5 snRNP200 surface expression. In comparing cell surface U5 snRNP200-high and -low AML cells (top and bottom 10% surface ADT expression, respectively), U5 snRNP200-high AML cells were characterized by significant enrichment of pathways responding to and mediating the inflammatory response (Fig. 7d). Interestingly, this prominently included upregulation of *IFITM2* and *IFITM3* (encoding interferon-induced transmembrane proteins 2 and 3) in U5 snRNP200-high AML cells (Fig. 7e). These data are potentially consistent with a prior report of U5 snRNP200 in the inflammatory response to viral RNA infections through activation of interferon-stimulated genes via the transcription factor complex ISGF3 (ref. ^35^). These results were further supported by mapping the AML blast cell compartment, which illustrated the presence of distinct proteogenomic subsets (Fig. 7f), consistent with a previous similar analysis of AML blast populations^31^. Application of a colorimetric ADT scale filtered to demonstrate the highest U5 snRNP200-surface expressing cells (top 10%) to this map revealed expression of surface U5 snRNP200 on the monocyte-like AML subset (Fig. 7g, left) and validated the corresponding upregulation of *IFITM2* and *IFITM3* gene expression on the same U5 snRNP200-high AML cells (Fig. 7g). Finally, unbiased proteogenomics via CITE-seq confirmed strong correlation of cell surface CD32 (*r* = 0.6183783, *P* < 2.2 × 10^16^) and CD33 (*r* = 0.3262483, *P* < 2.2 × 10^16^) with U5 snRNP200 among AML blasts with the highest (top 10%) U5 snRNP200 expression (Fig. 7g).

### In vivo efficacy of anti-U5 snRNP200 antibodies in AML models

**Fig. 8:**
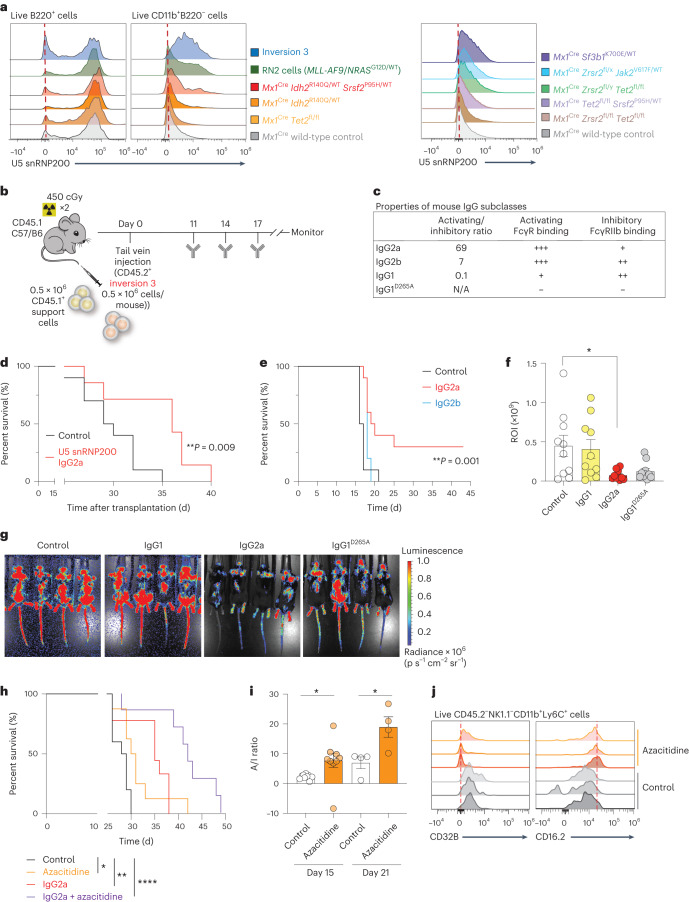
Anti-U5 snRNP200 antibodies with high affinity for FcγRs demonstrate robust anti-leukemic effects. **a**, Histogram overlays of U5 snRNP200 surface expression on peripheral blood B cells (left) and malignant myeloid cells (middle and right) by flow cytometry in murine genetically engineered models of AML. **b**, Schema of mouse inversion 3 AML model transplantation and treatment schedule. **c**, Table describing mouse IgG Fc subclass binding to activating and inhibitory Fc receptors. N/A, not applicable. **d**, Kaplan–Meier survival curve of recipient mice engrafted with inversion 3 AML cells following treatment with anti-U5 snRNP200 antibody engineered with the IgG2a Fc subclass (*n* = 7) or control (PBS) (*n* = 10). *P* values are from the log-rank test. **e**, Kaplan–Meier survival curve of recipient mice engrafted with mouse RN2 (*MLL-AF9* overexpression and *NRAS*^G12D^ mutation) cells following treatment with anti-U5 snRNP200 antibody engineered with IgG2a Fc, IgG2b Fc or control. *P* values are from the log-rank test. **P = 0.0012 (*n* = 10 mice per group). **f**, Quantification of bioluminescent imaging comparing RN2 disease burden on day 14 among control mice and mice treated with anti-U5 snRNP200 antibody (*n* = 10 mice per group). *P* values are from the unpaired *t*-test; *P = 0.0204. ROI, region of interest. **g**, Representative images of bioluminescent signal (measured on day 14) in control mice and mice treated with anti-U5 snRNP200 antibody. **h**, Kaplan–Meier survival curve of recipient mice engrafted with inversion 3 AML cells following treatment with anti-U5 snRNP200 antibody engineered with the IgG2a Fc subclass or control with or without concomitant azacitidine treatment. *P* values are from the log-rank test; *P = 0.0110, **P = 0.0035, ****P < 0.0001. **i**, Fc receptor activating/inhibitory (A/I) ratios (CD16.2/CD32B) on peripheral blood CD45.2^−^ monocytes or macrophages after 5 days of in vivo control or azacitidine treatment in inversion 3-engrafted mice (day 15, *n* = 9 mice per group; day 21, *n* = 4 mice per group). *P* values are from the unpaired *t*-test; left, *P = 0.036; right, *P = 0.023. **j**, Representative expression histograms from individual mice in **i**. Data are mean ± s.e.m. Source data

**Extended Data Fig. 4:**
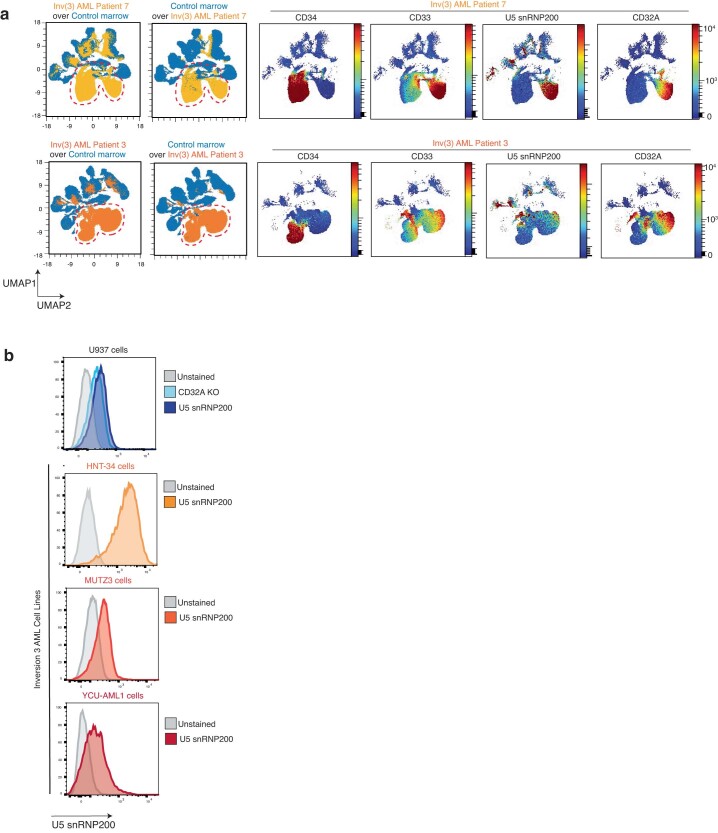
Cell surface U5 snRNP200 expression on inversion(3) AML patient samples and human patient-derived cell lines. **(a)** UMAP overlay comparing normal donor (blue islands) and AML samples for identification of malignant AML cells (top panel, yellow cell islands; bottom panel, orange cell islands) and colorimetric overlays of CD34, CD33, U5 snRNP200, and CD32A. **(b)** Histograms of U5 snRNP200 expression on U937 cells versus three distinct human inversion(3) AML cell lines (HNT-34, MUTZ3, and YCU-AML1).

The presence of U5 snRNP200 on the surface of AML cells and not on normal HSPCs highlights U5 snRNP200 as an attractive therapeutic target in AML. To investigate the anti-leukemic effects of U5 snRNP200 antibodies in syngeneic immunocompetent AML models, we first assessed surface U5 snRNP200 expression in murine models of AML and control wild-type C57/B6 mice. While we observed consistent U5 snRNP200 surface expression on B220^+^ B lymphocytes (Fig. 8a and Extended Data Fig. 3), as seen in humans, we observed a range of U5 snRNP200 surface expression on malignant myeloid cells across a number of myeloid leukemia mouse models (Fig. 8a). Across nine models, expression of U5 snRNP200 was most prominent on AML cells from mice bearing the humanized inversion chromosome 3q21q26 allele (‘inversion 3 mice’)^36,37^ as well as simultaneous overexpression of fusion gene *MLL-AF9* (also known as *KMT2A*-*MLLT3*) and *NRAS*^G12D^ cDNA (known as ‘RN2’ cells^38^). Evaluation of cell surface U5 snRNP200 expression on five *EVI1*(also known as *MECOM*)-rearranged AML patient samples by high-density 36-color spectral flow cytometry as well as on three human *EVI1*-rearranged patient-derived AML cell lines (HNT-34, MUTZ-3 and YCU-AML1) revealed clear U5 snRNP200 surface expression on all five *EVI1*-rearranged patient samples as well as an overlap with CD33 and CD32A expression (Extended Data Fig. 4 and Supplementary Table 4).

**Extended Data Fig. 5:**
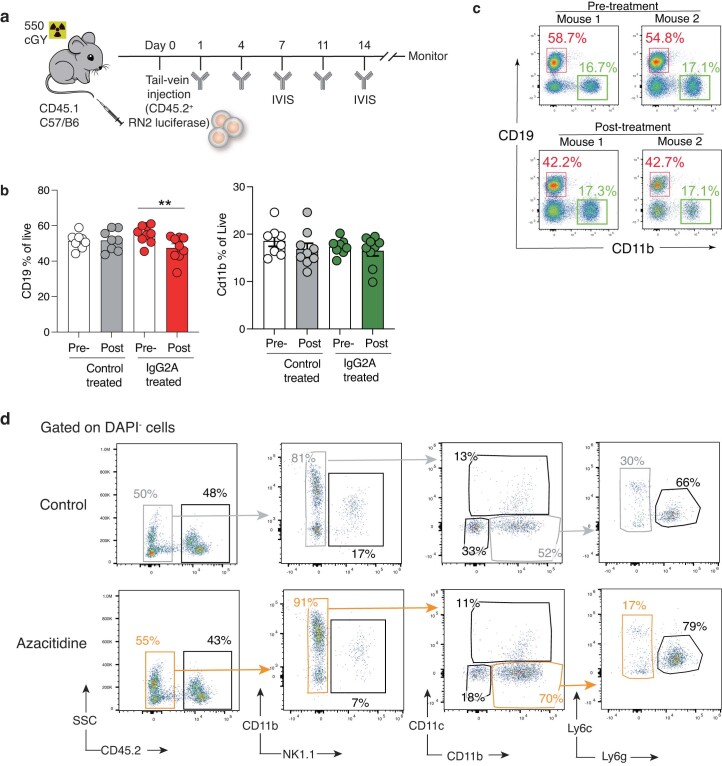
RN2 AML Model for U5 snRNP200 antibody treatment and assessment of azacitidine effects on hematopoietic cells. **(a)** Schematic summarizing RN2 cell engraftment, antibody treatment schedule, and disease burden assessment. **(b)** Impact of control or IgG2A anti-U5 snRNP200 antibody treatment on CD19 (left) and CD11b (right) cells in the peripheral blood. Bar graphs represent mean ± SEM shown. Control group n = 9 mice; treated group n = 10 mice. (p value from paired t-test; **p = 0.032. **(c)** Representative FACS plots of data from (b). **(d)** Gating schema for identification, quantification, and FcR expression characterization of immune cell subsets in CD45.1 mice engrafted with CD45.2 AML cells. From the live, CD45.2^−^ gate: NK cells = CD11b^−^NK1.1^+^, DCs = NK1.1^−^CD11C^+^, Monocyte/Macrophage = NK1.1^−^CD11c^−^CD11b^+^Ly6g^−^; Neutrophils (PMN) = NK1.1^−^CD11c^−^CD11b^+^Ly6g^+^. Source data

Using the inversion 3 mouse AML model (Fig. 8b), we tested an anti-U5 snRNP200 antibody variant with high affinity for activating Fc receptors on immune cell subsets (IgG2a; Fig. 8c). The single-agent IgG2a anti-U5 snRNP200 antibody yielded robust anti-leukemic activity, leading to a survival benefit compared to control mice treated with phosphate-buffered saline (PBS) (Fig. 8d). The fact that the IgG2a anti-U5 snRNP200 antibody provided therapeutic benefit suggests that the cellular mechanism of action of these antibodies may be via ADCC or antibody-dependent cellular phagocytosis^39^. To test this hypothesis, we next generated anti-U5 snRNP200 antibodies with murine subclass variant Fc regions that have lower activating/inhibitory ratios for Fc receptor engagement than the IgG2a variant (Fig. 8c). This included anti-U5 snRNP200 antibodies with murine IgG2b or IgG1 Fc regions as well as an engineered version of IgG1 with a D265A substitution that does not bind Fc receptors^40^. As we observed in the inversion 3 mouse model, in vivo treatment of animals engrafted with RN2 cells (Extended Data Fig. 5a) with the IgG2a variant antibody yielded a consistent survival benefit compared to the antibody variant IgG2b that had lower affinity for activating Fc receptors (Fig. 8e). Therapeutic benefit was also evaluated based on quantification of bioluminescent imaging (as RN2 cells also contain a luciferase vector) (Fig. 8f,g), which again supported IgG2a as the variant with the maximum anti-leukemic effect, as it provided significant disease control compared to variants that do not activate FcRs (IgG1 and IgG1^D265A^). Given the expression of U5 snRNP200 on the cell surface of normal B cells as well as subsets of mature NK cells and monocytes, we also evaluated the impact of treatment with anti-U5 snRNP200 antibodies on these normal cell populations in vivo. This revealed a clear (~14%) downregulation of the frequency of peripheral blood CD19^+^ cells following just two doses of the IgG2a anti-U5 snRNP200 antibody. By contrast, there was no consistent change in NK or CD11b^+^ cell frequency (Extended Data Fig. 5b,c).

**Extended Data Fig. 6:**
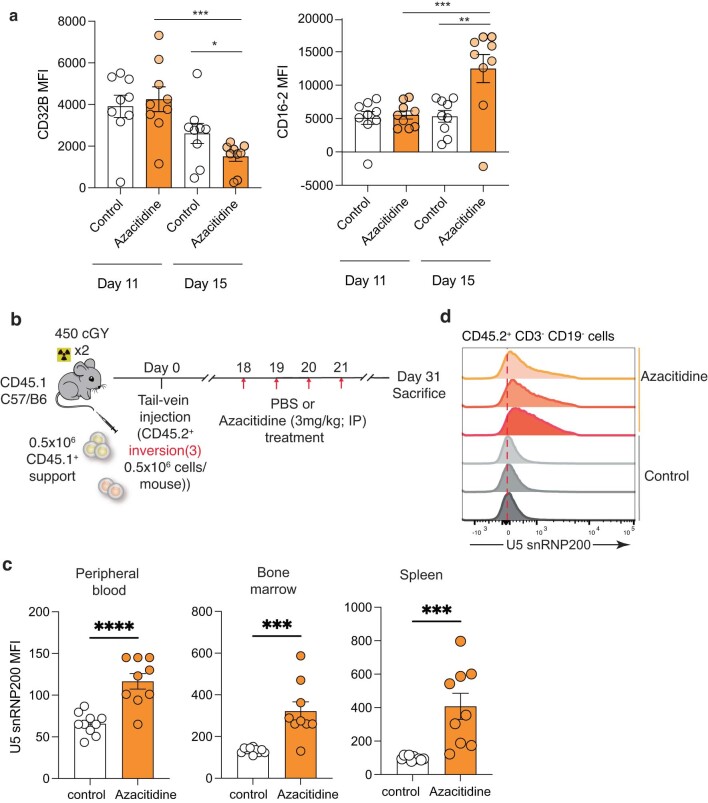
Impact of *in vivo* azacitidine treatment on Fc receptor expression on normal immune cells and U5 snRNP200 expression on AML cells. **(a)** Quantification of inhibitory FcR CD32B and activating FcR CD16.2 expression on monocytes/macrophages in peripheral blood from mice engrafted with inversion 3 AML after 5 days of control (PBS) versus azacitidine treatment. n = 9 mice per group. Bar graphs represent ± SEM (p from unpaired t-test; left *** p = 0.0005 and *p = 0.05; right *** p = 0.0007 and ** p = 0.007). FcR = Fc Receptor. **(b)** Schematic of experiment to test the impact of *in vivo* treatment expression on expression of U5 snRNP200 on the surface of AML cells. **(c)** Median fluorescence intensity of U5 snRNP200 expression on CD45.2^+^ malignant cells in peripheral blood (left), bone marrow (middle), spleen (right). n = 9 mice per group. Bar graphs represent ± SEM (p from unpaired t-test; left **** p = <.0001 middle ***p = 0.0004, right *** p = 0.0006). **(d)** Representative FACS plot of U5 snRNP200 cell surface expression on malignant myeloid (CD45.2^+^ CD3^−^ CD19^−^) bone marrow cells following in vivo treatment with control or azacitidine (as quantified in (c)). Source data

Given the limited clinical success with any single-agent therapy for overt AML, we also investigated the therapeutic impact of combining the IgG2a anti-U5 snRNP200 antibody with a commonly used therapeutic for patients with AML: the nucleoside analog azacitidine. Azacitidine is currently being combined with other antibody-based therapeutic approaches in AML including anti-CD47 and anti-TIM-3 antibodies^2^, which provided further motivation for testing in combination with anti-U5 small nuclear ribonucleoprotein (snRNP) antibodies. Importantly, combined azacitidine and anti-U5 snRNP200 antibody treatment provided greater survival benefit to recipient mice engrafted with inversion 3 cells than either agent alone or the control (Fig. 8h). To interrogate the possible mechanism underlying the superior outcome of the combination therapy group, we profiled Fc receptor expression on immune cells in response to in vivo azacitidine treatment. Interestingly, azacitidine treatment was associated with increased activating Fc receptor CD16.2 (also known as FcγRIV) but also decreased inhibitory Fc receptor CD32B on monocytes and macrophages (CD45.2^−^NK1.1^−^CD11b^+^Ly6c^+^) that resulted in statistically significant improvement in the ratio of activating to inhibitory receptor expression (Fig. 8i,j and Extended Data Figs. 5d and 6a). Moreover, evaluation of cell surface U5 snRNP200 on CD45.2^+^ inversion 3 murine AML cells engrafted into CD45.1^+^ recipient mice following azacitidine treatment revealed significant cell surface U5 snRNP200 upregulation on malignant cells in peripheral blood, bone marrow and spleen 10 d following the last dose of azacitidine (Extended Data Fig. 6b–d).

These results support U5 snRNP200 targeting as a promising therapeutic for AML, identify that anti-U5 snRNP200 antibodies exert maximum therapeutic benefit via activation of FcRs and highlight a contribution of azacitidine to improving the balance of activating/inhibitory Fc receptors in the AML microenvironment as well as impact on AML cell surface U5 snRNP200 abundance.

## Discussion

In this study, a high-parameter spectral flow cytometry approach in conjunction with proteogenomic assessment by CITE-seq demonstrated expression of U5 snRNP200, a highly conserved component of the RNA spliceosome, on the surface of malignant AML cells but not primitive hematopoietic stem or progenitor cells. Knock-in of the sequence for an epitope tag into the locus of the gene encoding U5 snRNP200 further rigorously confirmed membrane localization of U5 snRNP200, above and beyond the use of anti-U5 snRNP200 antibodies (which could be recognizing other proteins with domains homologous to those in U5 snRNP200). This differential expression makes U5 snRNP200 an attractive therapeutic antibody target as it circumvents the on-target off-tumor side effects that are a liability of the majority of antigen targets currently pursued for AML therapy. While U5 snRNP200 is expressed on B cells and a subset of NK cells and monocytes, this expression pattern is far more limited than therapeutic targets being currently explored for AML such as CD47 or CD123. Furthermore, we define patterns of antigens coexpressed with U5 snRNP200 within the malignant AML compartment that may be suitable for targeting via a multispecific or bispecific antibody or multi-antigen CAR T cell therapy.

U5 snRNP200 is a conserved and essential component of the RNA splicing machinery, which is a nuclear enzymatic process that does not clearly involve cytoplasmic- or cell membrane-localized processes. Interestingly, however, prior data demonstrated that the U5 snRNP complex forms in the cytoplasm specifically without U5 snRNP200, which later assembles into the larger U5 snRNP complex within the nucleus^41^. These data suggested a potential cytoplasmic role for U5 snRNP200. More recently, a prior study identified an immunoregulatory role of cytoplasmic U5 snRNP200 as a viral RNA sensor and TBK1 adaptor required for activation of the iIRF3-mediated antiviral innate response^42^. Here, single-cell RNA-seq in AML cells revealed a striking association between cell surface U5 snRNP200 and expression of factors within the same antiviral innate response pathway. Whether the RNA-binding or helicase function of cell membrane-localized U5 snRNP200 plays a role in AML pathogenesis will be interesting to explore and could provide another therapeutic, as chemical inhibitors of U5 snRNP200 helicase activity have been developed^43^.

In addition to exploring therapeutic antigen targets in AML, we also provide a clear delineation of human FcγR expression on immune cells within control donor and AML bone marrow microenvironments, a critical factor for effective antibody therapy. Specifically, we identify increased expression of the inhibitory CD32B receptor on immune effector cell populations within the bone marrow of patients with AML, therefore unveiling a previously unappreciated explanation for limited responses to therapeutic antibodies in the treatment of AML. These findings support the development of antibodies engineered to specifically bind activating FcγRs with absent or minimal binding to the inhibitory FcγR, a point demonstrated here by the evaluation of multiple classes of anti-U5 snRNP200 antibodies with distinct FcγR engagement.

Despite the evidence supporting immune-mediated clearance in response to IgG2a anti-U5 snRNP200 antibodies, the engineered anti-U5 snRNP200 antibody, unable to bind any FcγRs (IgG1^D265A^), which serves as a true blocking antibody, also resulted in a trend toward disease control. These data suggest a possible anti-leukemic contribution of U5 snRNP200-targeting antibodies by blocking signaling through cell surface U5 snRNP200. Given the tight association we identify here of cell surface U5 snRNP200 and CD32A, a well-characterized activating FcR in which signaling through its ITAM domain activates mitogenic signaling pathways, it will be interesting to explore whether U5 snRNP200 interaction with CD32A promotes pathologic signaling in AML.

Overall, the studies reported here not only provide a high-density map of AML-associated antigens and distribution of Fc receptor expression, which have the potential for immediate development of antibody-based therapies and rationally designed combination approaches for AML, but also capture previously unknown aspects of AML disease biology that may determine response to antibody therapy.

## Methods

This study complies with all relevant ethical regulations. Studies involving patient samples were approved by the institutional review boards of Memorial Sloan Kettering Cancer Center (MSKCC) and conducted in accordance with the Declaration of Helsinki protocol. Specimens were obtained as part of the MSKCC Institutional Review Board-approved clinical protocol 06-107 to which all participants consented. O.A-W. is a participating investigator on this protocol. All animal procedures were completed in accordance with the Guidelines for the Care and Use of Laboratory Animals and were approved by the Institutional Animal Care and Use Committees at MSKCC. All mouse experiments were performed in accordance with a protocol approved by the MSKCC Institutional Animal Care and Use Committees (13-04-003).

### Cell lines and cell culture

HEK293T were obtained from the American Type Culture Collection (ATCC, CRL-3216) and cultured in DMEM medium with 10% FBS. HNT-34 (purchased from DSMZ, ACC 600) and 5637 (purchased from ATCC, HTB-9) cells were cultured in RPMI 1640 with 10% FBS. MUTZ-3 cells (purchased from DSMZ, ACC 295) were cultured in a minimum essential medium (with ribonucleosides and deoxyribonucleosides) with 20% FBS and 20% conditioned medium from the cell line 5637. YCU-AML1 cells (gift from H. Nakajima, Yokohama City University) were cultured with OP-9 (purchased from ATCC, CRL-2749) in IMDM medium with 10% FBS, 55 mM β-mercaptoethanol (Sigma-Aldrich) and 20 ng ml^−1^ granulocyte–macrophage colony-stimulating factor (PeproTech). U937 wild-type (CRL-1593.2, ATCC) and U937 *FCGR2A*-knockout cells were generated previously^44^, and CD32A re-expression was achieved by introducing the full-length sequence or a version with in-frame deletion of the sequence for the transmembrane domain cloned into the piggyBac vector. Murine RN2 cells (*MLL-AF9*, *NRAS*^G12D^) were generated as previously described^45^ and cultured in RPMI medium with 10% FBS and 1% penicillin–streptomycin and passaged every 2–3 d to maintain a density of less than 1 × 10^6^ cells per ml. No further authentications were performed. No commonly misidentified cell lines were used in the study.

### Animals

Male and female 8–10-week-old CD45.1^+^ mice were purchased from Jackson Laboratory and maintained until the age of 12 weeks before use for transplantation studies. The inv(3)(3q21q26) mouse strain^36^ (RBRC09508) was provided by RIKEN BRC through the National BioResource Project of the MEXT and AMED, Japan. Male and female *Mx1*^Cre^ *Sf3b1*^K700E/WT^ inv(3)(q21q26) CD45.2^+^ cells^37^ were serially transplanted into male and female CD45.1^+^ mice. Mice were bred and maintained in individual ventilated cages and fed with autoclaved food and water at the Memorial Sloan Kettering Animal Facility. After transplantation, mice were maintained on acidified water and monitored closely for signs of disease or morbidity daily (or more frequently as required) for failure to thrive, weight loss > 10% total body weight, open skin lesions, bleeding, infection or fatigue. If mice developed any of the above complications or manifested symptoms of leukemia that were sufficiently informative (as assessed by blood count, failure to thrive, weight loss > 10% total body weight, open skin lesions, bleeding, infection and/or fatigue), they were killed immediately.

### Human patient samples

De-identified, clinically annotated primary human AML samples derived from bone marrow mononuclear cells were used. Mutational genotyping of each sample was performed by the MSK-IMPACT assay as described previously^46^. Bone marrow from unaffected donors was acquired from Stemcell Technologies. Informed consent was obtained from all participants before sample acquisition. Normal human viably frozen cell samples were obtained from commercial sources (Lonza, ATCC and Gibco).

### Generation of recombinant Fc receptor engineered antibodies

Sequences for anti-human snRNP200 (clone 1223, cross-reactive to both mouse and human U5 snRNP200) heavy and light chain variable regions were obtained from the patent literature and subsequently cloned into expression constructs for the various murine subclass variants (for example, mIgG1, mIgG1^D265A^, mIgG2b, mIgG2b, hIgG1) as previously described^40^. The variable heavy chain is CAGGTGCAGCTGGTGGAGTCTGGGGGAGGCGTGGTCCAGCCTGGGAGGTCCCTGAGACTCTCCTGTGCAGCGTCTGGATTCACCTTCAGTACCTATGGCATGCACTGGGTCCGCCAGGCTCCAGGCAAGGGGCTTGAGTGGGTGGCAGTTATATGGTATGATGGAAGTAATACATACTATGCAGACTCCGTGAAGGGCCGATTCACCATCTCCAGAGACAATTCCAAGAACACACTGTATCTGCAAATAAAGAGCCTGAGAGCCGAGGACACGGCTGTCTATTACTGTGCGAGAGGCCGTGGATATAGTGCCCAAGGGAATCGGAATAGGGCTTACTACTTTGACTACTGGGGCCAGGGAACCCTGGTCACCGTCTCCTCA. The variable light chain is TCTTCTGAGCTGACTCAGGACCCTGCTGTGTCTGTGGCCTTGGGACAGACAGTCAGGATCACATGCCAAGGAGACTTCCTCAGAAGCTATTATGCAAGCTGGTACCAGCAGAAGCCAGGACAGGCCCCTGTACTTGTCATCTTTGGTAAAAACAAGCGGCCCTCAGGGATCCCAGACCGATTCTCTGGCTCCAGCTCAGGAAACACAGCTTCCTTGACCATCACTGGGGCTCAGGCGGAAGATGAGGCTGACTATTACTGTAACTCCCGGGACCGCAGTGGTAACCACCTGGTGTTCGGCGGAGGGACCAAGCTGACCGTCCTA. Recombinant antibodies were generated by transient transfection of Expi293 cells with heavy and light chain-expression plasmids using previously described protocols. Before transfection, plasmid sequences were validated by direct sequencing (GENEWIZ). Recombinant IgG antibodies were purified from cell-free supernatants by affinity purification using protein G or protein A Sepharose beads (GE Healthcare). Purified proteins were dialyzed in PBS, filter sterilized (0.22 μm) and stored at 4 °C.

### Genome-scale CRISPR–Cas9 screening

The GFP^+^ lentivirus (U6 promoter, driving expression of sgRNA and RPBSA promoter, driving puromycin resistance and ZsGreen) carrying the genome-wide human Brunello library (77,441 sgRNA species targeting 19,114 genes and 1,000 nontargeting control sgRNA species) was produced in 293T cells. The viral titer was determined by measuring the percentage of puromycin-resistant cells following transduction. A titer resulting in approximately 30% transduction efficiency (puromycin resistant) was used for the following experiments to ensure only one viral integration per cell. U937 and K562 cells expressing Cas9 were transduced with Brunello lentivirus, and puromycin selection (8 µg ml^−1^ for U937 cells or 4 µg ml^−1^ for K562 cells) was performed for 2 d before flow cytometry (Aria, BD Biosciences) for GFP^+^ cells. After an additional 8 d in culture, GFP^+^ cells were stained with APC-labeled anti-U5 snRNP200 antibody and subjected to flow cytometry, during which the bottom and top 10% U5 snRNP200-expressing cell populations were collected. Cell pellets from each population were lysed, and genomic DNA was extracted using the QIAamp DNA Mini Kit (Qiagen) and quantified with the Qubit machine (Thermo Scientific). gRNA amplicons were amplified by PCR using TaKaRa Ex Taq DNA Polymerase (Takara) to add Illumina sequencing adaptors and multiplexing barcodes. Amplicons were quantified with the Qubit machine and the Bioanalyzer (Agilent), multiplexed and sequenced on an Illumina NextSeq 500 to obtain 75-bp single-end reads. Demultiplexed FASTQ files were trimmed from both the 5′ and the 3′ end to remove sequencing adaptor- and sgRNA-derived sequences using cutadapt (version 2.5) to yield the 20-nucleotide sequence of the sgRNA using the following parameters: ‘-g TGTGGAAAGGACGAAACACCG -a GTTTTAGAGCTAGAAATAGCAAG–maximum-length 20’. Next, the frequency of each sgRNA was determined using the ‘count’ function of the MAGeCK (version 0.5.9.4) software package. sgRNA counts were normalized to sequencing depth by applying the following parameters: ‘–norm-method total’. The corresponding normalized count matrix was used to perform the indicated pairwise statistical comparisons using the ‘test’ function of the MAGeCK package. All visualization of these CRISPR–Cas9 screening data was performed in RStudio (version 1.3.1073) using the ggplot2 (version 3.3.5) package. Functional and pathway enrichment against existing GO signatures was performed using ToppFun, part of the ToppGene Suite, and included GO terms and pathways from the KEGG, Reactome and BioCarta databases. GO terms or pathways were identified as significant under a Benjamini–Hochberg multiple-correction procedure at a false discovery-rate (FDR) cutoff of 0.05. As input for ToppFun, we manually selected the top 150 enriched genes (ranked by FDR) as identified by MAGeCK. GO term data were plotted in GraphPad Prism 8.

### Western blotting

K562 cells expressing HaloTagged U5 snRNP200, parental K562 cells and *FCGR2A*-knockout K562 cells were collected by centrifugation, and subcellular fractions were obtained using a subcellular protein fractionation kit (Thermo Fisher). Protein concentrations were measured with the BCA reagent, and 10 µg was loaded per lane onto 4–12% Bis-Tris protein gels. After transfer, PVDF membranes were probed with anti-snRNP200 antibody (Bethyl Laboratories), anti-HaloTag antibody (Promega), anti-CD32A antibody (R&D Systems), anti-sodium–potassium ATPase antibody (Cell Signaling Technologies), anti-tubulin antibody (Cell Signaling Technologies) or anti-SP1 antibody (Cell Signaling Technologies), followed by appropriate peroxidase-conjugated secondary antibodies, and visualized as previously described.

### Flow cytometry

For conventional flow cytometry experiments, cells were collected by centrifugation and washed once with cold PBS before application of Fixable LIVE/DEAD NIR (Thermo Fisher). Mouse or human Fc receptor blocking was applied before staining (for all experiments in which Fc receptors were not profiled individually) with an antibody cocktail containing Brilliant Stain Buffer (BD Biosciences) and monocyte blocker (BioLegend). After cocktail staining, cells were washed twice with cold PBS before data acquisition using the Attune cytometer (Thermo Fisher), and analysis was performed using FlowJo software (version 9.0) or FACSDiva software (version 9.0). The 36-color or -parameter spectral flow cytometry panel was developed with guidance from Cytek Biosciences and a recently published 40-color peripheral blood spectral flow cytometry panel (OMIP-069)^47^, including individual antibody titrations and comparisons of performance in single versus multicolor staining. For spectral flow cytometry experiments, human bone marrow samples were thawed using prewarmed BD BSA stain buffer (BD Biosciences), washed twice with ice-cold buffer and counted. After application of Fixable LIVE/DEAD NIR (diluted 1:3,000 in PBS; Thermo Fisher) to a maximum of 5 million cells, sequential staining included Fc receptor antibody cocktail containing Brilliant Stain Buffer and monocyte blocker followed by anti-CXCR5 antibody and finally the remaining surface antibody panel cocktail. Before acquisition using the Aurora cytometer (Cytek), samples were washed twice with cold BD BSA stain buffer. All antibody information including clones, vendors and dilution factors is summarized in Supplementary Table 1. Post-acquisition unmixing was performed using SpectroFlo software version 3.0 (Cytek). Analysis of samples including scaling, data-cleanup gating, manual gating, UMAP generation and heatmap generation was conducted using OMIQ software (online platform).

### Cellular indexing of transcriptomes and epitopes by sequencing

Human bone marrow samples were thawed using prewarmed RPMI with 10% FBS and washed twice with cold PBS before labeling with unique TotalSeqC-compatible HashTags (BioLegend). Samples were subsequently incubated with human Fc receptor-blocking agent (BioLegend) and labeled with DAPI for live cell sorting by flow cytometry using an Aria cytometer (BD Biosciences). After sorting, 150,000 cells were washed once with cold PBS before application of the TotalSeqC cocktail (BioLegend) and subsequently a custom ADT-tagged anti-U5 snRNP200 antibody (BioLegend). Cells were washed three times with cold PBS and submitted to the MSKCC Integrative Genomics Core for sequencing.

FASTQ reads were processed using the Cell Ranger version 7.0.0 ‘count’ workflow to generate gene expression and antibody capture data matrices; the Cell Ranger ‘multi’ pipeline was additionally executed to demultiplex hashed sequencing samples. Resultant filtered sparse count matrices were loaded into R version 4.0.0 as Seurat version 4.0.6 objects. Multiplets were tagged using scDblFinder version 1.10.0 and removed. Further filtering was applied to only retain (1) RNA features detected in more than three cells and (2) cells with more than 200 and less than 2,500 detected features. Gene expression and antibody capture data were normalized using Seurat’s ‘LogNormalize’ and DSB’s (version 1.0.2) ‘DSBNormalizeProtein’ functions, respectively. Corrected data (*n* = 42,251) were used to perform clustering and generate a multimodal UMAP using Seurat’s weighted nearest-neighbor workflow.

To determine cluster identities, an annotated reference of AML (at-diagnosis) and healthy control bone marrow aspirates was loaded into R (*n* = 22 samples, *n* = 22,600 cells). Thereafter, blast and normal cell type markers were identified using SingleR’s (version 1.4.1) ‘trainSingleR’ function and with the differential expression method set to the Wilcoxon ranked-sum test. Reference-defined labels were then applied to experimental data using Singler’s ‘classifySingleR’ function on log-normalized read counts. Putative labels were manually validated by examining canonical marker expression and by performing pseudotime analysis using Monocle version 2.24.1.

Differential gene expression analysis between subsets of interests was performed using Seurat’s ‘findMarkers’ function (Wilcoxon ranked-sum test), and subsequent GSEA in fgsea version 1.22.0 against hallmark gene sets was performed using normalized RNA data.

### Bone marrow transplantation

Freshly dissected femora and tibiae were isolated from CD45.1^+^ WT and CD45.2^+^ *Mx1*^Cre^ *Sf3b1*^K700E/WT^ inv(3)(q21q26) mice. Bones were spun at 300*g* by benchtop centrifugation, and RBCs were lysed in ammonium chloride–potassium bicarbonate lysis buffer for 5 min. After centrifugation, cells were resuspended in ice-cold sterile PBS, passed through a 100-μm cell strainer and counted. Finally, a total of 0.5 million bone marrow cells from CD45.2^+^ *Mx1*^Cre^ *Sf3b1*^K700E/WT^ inv(3)(q21q26) mice were mixed with 0.5 million wild-type CD45.1^+^ support bone marrow cells and transplanted by tail vein injection into lethally irradiated (two times, 450 cGy) CD45.1^+^ recipient mice. Engraftment was measured by flow cytometry from the peripheral blood 10 d after transplantation. For syngeneic RN2 cell-transplantation experiments, 50,000 cells were injected into sublethally irradiated (550 cGy) CD45.1^+^ recipient mice.

### Animal antibody treatments

Mice engrafted with RN2 or *EVI1*-rearranged *Mx1*^Cre^ *Sf3b1*^K700E/WT^ AML were treated with a 400-µl intraperitoneal (i.p.) injection of control (PBS) or antibody (1 mg ml^−1^) according to the indicated experiment schemas. Mice were randomized to all treatments, and data collection from animals was performed in a randomized fashion. Azacitidine was dissolved in 20% 2-hydroxypropyl-β-cyclodextrin in sterile PBS and was dosed for 3 d (days 1–3, RN2 model) or 5 d (days 10–14, *EVI1* model) at 3 mg per kg by i.p. injection. All whole-body bioluminescent imaging was performed by i.p. injection of luciferin (GoldBio) at a concentration of 50 mg per kg, and imaging was performed after a 5-min incubation with the IVIS system. Bioluminescent signals (radiance) were quantified using Living Image software with standard region-of-interest rectangles.

### Statistics and reproducibility

No statistical methods were used to predetermine sample sizes, but our sample sizes are similar to those reported in previous publications^48,49^, and the experiments were not randomized. The investigators were not blinded to allocation during experiments and outcome assessments. Data collection and analysis were not performed blind to the conditions of the experiments. No data were excluded from the analyses. Data distribution was assumed to be normal, but this was not formally tested. Bar graphs are presented as mean ± s.e.m. For statistical comparisons between experimental groups, ANOVA (when multiple groups were compared simultaneously), followed by either the Mann–Whitney test or the unpaired *t*-test with Welch’s correction was applied based on distribution of data values, and Bonferroni’s correction for multiple comparisons was applied when applicable (when statistically significant effects were found). Statistical differences between survival rates were analyzed by comparison of Kaplan–Meier curves using the log-rank (Mantel–Cox) test. Statistical analyses were performed using Prism software (GraphPad version 10.0.0). Data with statistical significance are as indicated. Further information on research design is available in the Nature Portfolio Reporting Summary linked to this article.

### Immunoprecipitation–mass spectrometry

Cells were fractionated as described above before overnight incubation at 4 °C with protein A agarose beads (Millipore) conjugated to anti-CD32A antibody (clone IV.3) resuspended in immunoprecipitation lysis buffer (Pierce). Beads were subsequently washed three times using immunoprecipitation lysis buffer and three times using 10 mM Tris-HCl, 150 mM NaCl, pH 7.5.

### Protein digestion for proteomic analyses

Beads were resuspended in 40 µl of 2 M urea, 50 mM ammonium bicarbonate, pH 8.5 and treated with dl-dithiothreitol (final concentration, 1 mM) for 30 min at 37 °C with shaking (1,100 r.p.m.) on a ThermoMixer (Thermo Fisher). Free cysteine residues were alkylated with 2-iodoacetamide (final concentration, 3.67 mM) for 45 min at 25 °C and 1,100 r.p.m. in the dark. LysC (750 ng) was added, followed by incubation for 1 h at 37 °C and 1,150 r.p.m. Finally, trypsin (750 ng) was added, followed by incubation for 16 h at 37 °C and 1,150 r.p.m.

After incubation, the digest was acidified to pH <3 with the addition of 50% trifluoroacetic acid, and the peptides were desalted on 3-plug C18 (3M Empore High Performance Extraction Disks) stage tips. Briefly, the stage tips were conditioned by sequential addition of (1) 100 μl 100% acetonitrile (ACN), (2) 100 μl 70% ACN–0.1% trifluoroacetic acid, (3) 100 μl 0.1% formic acid, (4) 100 μl 0.1% formic acid. Following conditioning, the acidified peptide digest was loaded onto the stage tip. The stationary phase was washed once with 100 μl 0.1% formic acid. Finally, samples were eluted using 50 μl of 70% ACN–0.1% formic acid twice. Eluted peptides were dried under vacuum, followed by reconstitution in 12 μl of 0.1% formic acid, sonication and transfer to an autosampler vial. Peptide yield was quantified with the NanoDrop (Thermo Fisher).

### Mass spectrometry analyses

Peptides were separated on a 50-cm column composed of C18 stationary phase (Thermo Fisher, ES903) using a gradient from 0.5% to 25% buffer B over 100 min, to 50% in 15 min and to 90% in 5 min (buffer A, 0.1% formic acid in HPLC-grade water; buffer B, 99.9% ACN, 0.1% formic acid) with a flow rate of 300 nl min^−1^ using a nanoACQUITY HPLC system (Waters). Mass spectrometry data were acquired on an Eclipse mass spectrometer (Thermo Fisher Scientific) using a data-independent acquisition method. The method consisted of one MS1 scan, standard AGC target, a maximum injection time of 50 ms, a scan range of 380–985 *m*/*z* and a resolution of 120,000. Fragment ions were analyzed in 60 data-independent acquisition windows at a resolution of 15,000.

### Data-independent acquisition data analysis

Raw data files were processed using Spectronaut version 17.4 (Biognosys) and searched with the Pulsar search engine with a *Homo sapiens* UniProt protein database downloaded on 23 September 2022 (226,953 entries). Cysteine carbamidomethylation was specified as a fixed modification, while methionine oxidation, acetylation of the protein N terminus and deamidation (NQ) were set as variable modifications. A maximum of two trypsin-missed cleavages were permitted. Searches used a reversed sequence decoy strategy to control the peptide FDR, and 1% FDR was set as the threshold for identification. The unpaired *t*-test was used to calculate *P* values in differential analysis; the volcano plot was generated based on log_2_ (fold change) and *q* values (multiple-testing-corrected *P* values). *A q* value of ≤0.05 was considered the statistically significant cutoff.

### Reporting summary

Further information on research design is available in the Nature Portfolio Reporting Summary linked to this article.

### Supplementary information

Reporting Summary

Supplementary Tables Supplementary Tables 1–4.

### Source data

Source Data Fig. 1 Statistical source data for Figs. 1–8 and Extended Data Figs. 1–3, 5 and 6.

Source Data Fig. 4 Unprocessed western blots and gels.

Source Data Fig. 6 Unprocessed western blots and gels.

## Data Availability

CITE-seq and AML cell line CRISPR screen data have been deposited under Gene Expressiom Omnibus (GEO) accession GSE220474. Immunoprecipitation–mass spectrometry data have been deposited at the ProteomeXchange Consortium via the PRIDE partner repository with the dataset identifier PXD042514 located at this page: http://www.ebi.ac.uk/pride/archive/projects/PXD042514. The datasets used in this study were UniProt (https://www.uniprot.org/) and a previously published reference dataset of bone marrow samples from newly diagnosed patients with AML and healthy age-matched controls at GSE116256 (ref. ^31^). All other data supporting the findings of this study are available from the corresponding author upon request. Source data are provided with this paper.

## Code availability

All packages used for the bioinformatic analysis are described in the Methods.
