# Supplementary material for: Systematic evaluation of AML-associated antigens identifies anti-U5 SNRNP200 therapeutic antibodies for the treatment of acute myeloid leukemia
Source: Nat Cancer. 2023 Oct 23;4(12):1675–92. doi: 10.1038/s43018-023-00656-2 (PMC10733148; doi:10.1038/s43018-023-00656-2)
Supplement: Supplementary file 1 — Reporting Summary [file 43018_2023_656_MOESM1_ESM.pdf]

nature portfolio

Corresponding author(s): Jeffrey Ravetch and Omar Abdel-Wahab

Last updated by author(s): Jul 26, 2023

Reporting Summary

Nature Portfolio wishes to improve the reproducibility of the work that we publish. This form provides structure for consistency and transparency in reporting. For further information on Nature Portfolio policies, see our Editorial Policies and the Editorial Policy Checklist.

Statistics

For all statistical analyses, confirm that the following items are present in the figure legend, table legend, main text, or Methods section.

n/a	Confirmed	
☐	☒	The exact sample size (n) for each experimental group/condition, given as a discrete number and unit of measurement
☐	☒	A statement on whether measurements were taken from distinct samples or whether the same sample was measured repeatedly
☐	☒	The statistical test(s) used AND whether they are one- or two-sided<br>*Only common tests should be described solely by name; describe more complex techniques in the Methods section.*
☐	☒	A description of all covariates tested
☐	☒	A description of any assumptions or corrections, such as tests of normality and adjustment for multiple comparisons
☐	☒	A full description of the statistical parameters including central tendency (e.g. means) or other basic estimates (e.g. regression coefficient) AND variation (e.g. standard deviation) or associated estimates of uncertainty (e.g. confidence intervals)
☐	☒	For null hypothesis testing, the test statistic (e.g. F, t, r) with confidence intervals, effect sizes, degrees of freedom and P value noted<br>*Give P values as exact values whenever suitable.*
☒	☐	For Bayesian analysis, information on the choice of priors and Markov chain Monte Carlo settings
☒	☐	For hierarchical and complex designs, identification of the appropriate level for tests and full reporting of outcomes
☐	☒	Estimates of effect sizes (e.g. Cohen's d, Pearson's r), indicating how they were calculated

Our web collection on statistics for biologists contains articles on many of the points above.

Software and code

Policy information about availability of computer code

Data collection	SpectroFlow Flow Cytometry Software (Cytek Bioscience), Attune NxT Flow Cytometry Software (Thermo Fischer Scientific), FACS Diva Software flow cytometry sorting (BD Biosciences), Living Image (IVIS), Microsoft Excel 2008
Data analysis	Prism (GraphPad v10.0.0), OMIQ (online platform), Cell Ranger v7.0.0, scDblFinder v1.10.0, Seurat, SingleR v1.4.1, Monocle v2.24.1, GSEA v1.22.0, FlowJo v.9.0, FACS Diva v9.0, ggplot2 (v3.3.5) package.

For manuscripts utilizing custom algorithms or software that are central to the research but not yet described in published literature, software must be made available to editors and reviewers. We strongly encourage code deposition in a community repository (e.g. GitHub). See the Nature Portfolio guidelines for submitting code & software for further information.

Data

Policy information about availability of data

All manuscripts must include a data availability statement. This statement should provide the following information, where applicable:

- Accession codes, unique identifiers, or web links for publicly available datasets
- A description of any restrictions on data availability
- For clinical datasets or third party data, please ensure that the statement adheres to our policy

The CITE-seq and AML cell line CRISPR screen data have been deposited under GEO accession ID GSE 220474. The immunoprecipitation mass spectrometry data have been deposited to the ProteomeXchange Consortium via the PRIDE partner repository with the dataset identifier PXD042514 located at this page: http://

www.ebi.ac.uk/pride/archive/projects/PXD042514
The datasets used in this study were Uniprot, https://www.uniprot.org/ and a previously published reference dataset of newly diagnosed AML and healthy age-matched control bone marrow samples in GSE116256.31 Source data for Fig. 1-5 and Extended Data Fig. 1-8 have been provided as Source Data files. All other data supporting the findings of this study are available from the corresponding author upon request.

Human research participants

Policy information about studies involving human research participants and Sex and Gender in Research.

Reporting on sex and gender	The term sex is applied to patient samples to indicate biologic attribute throughout all studies performed.
Population characteristics	The studies utilize bone marrow sample from 46 newly diagnosed AML patients. This cohort represents the heterogeneous range of disease features in newly diagnosed AML patients with a median age of 58 years and with 20% and 65% of patients being of intermediate or adverse risk, respectively, according to 2022 European LeukemiaNet risk classification. The clinical parameters including age, sex, risk stratification, and mutation status are summarized in figure 1a.
Recruitment	All samples were collected prospectively on MSKCC protocol 06-107 and used under MSK protocol 16-171. All patients with AML 18 years of age or older are eligible for this study. Protocols 06-107 and 16-171 were most recently approved by the Human Subjects Protection Committee at the Memorial Sloan Kettering Cancer Center on June 28, 2022 and February 22, 2022, respectively and are fully HIPAA compliant. Informed consent for participation is obtained from each subject. This study does not exclude any individuals on the basis of sex or gender or ethnicity or race. Every effort is made to encourage participation by women as well as men and by ethnic and racial minorities. Samples were chosen based on lack of prior treatment status for AML and with no bias on any other criteria of patient characteristics or any known self-selection bias. Eligibility criteria and registration requirements are closely monitored and supervised. All clinical investigators, including Dr. Abdel-Wahab, have obtained training in the ethical conduct of human research and have received appropriate certification of this training.
Ethics oversight	Memorial Sloan Kettering Cancer Center Institutional Review Board

Note that full information on the approval of the study protocol must also be provided in the manuscript.

Field-specific reporting

Please select the one below that is the best fit for your research. If you are not sure, read the appropriate sections before making your selection.

☒ Life sciences ☐ Behavioural & social sciences ☐ Ecological, evolutionary & environmental sciences

For a reference copy of the document with all sections, see nature.com/documents/nr-reporting-summary-flat.pdf

Life sciences study design

All studies must disclose on these points even when the disclosure is negative.

Sample size	With 10 mice in each group, we have 80% power to detect a 20% difference in cell populations assuming an effect size ($\Delta\mu/\sigma$) of 2.0 between groups using a Wilcoxon rank-sum test at the 0.05 significance level.
Data exclusions	No data exclusions.
Replication	In vivo assays were done in biological triplicate to assess reproducibility with 10 mice per group in each arm of each experiment. All attempts at replication were successful and all in vitro studies were performed in at least biological triplicate.
Randomization	Mice were assigned to treatment groups for in vivo studies by cage. No specific randomization strategy was used. All available newly diagnosed AML patient samples available, as well as AML cell lines, were profiled for cell surface antigen expression without allocation to groups.
Blinding	Investigators performing in vivo treatments were blinded to treatment type/group (i.e. control versus antibody).

Reporting for specific materials, systems and methods

We require information from authors about some types of materials, experimental systems and methods used in many studies. Here, indicate whether each material, system or method listed is relevant to your study. If you are not sure if a list item applies to your research, read the appropriate section before selecting a response.

Materials & experimental systems

n/a	Involved in the study
	☒ Antibodies
	☒ Eukaryotic cell lines
☒	Palaeontology and archaeology
	☒ Animals and other organisms
☒	Clinical data
☒	Dual use research of concern

Methods

n/a	Involved in the study
☒	ChIP-seq
	☒ Flow cytometry
☒	MRI-based neuroimaging

Antibodies

Antibodies used	Western blotting antibodies (diluted 1:1000 in 5% bovine serum albumin in PBS): human anti-snRNP200 (Bethyl Laboratories, rabbit poly-clonal, cat # 50-156-5174), anti-HaloTag (Promega clone not indicated cat #G9211), human anti-sodium-potassium ATPase (Cell Signaling Technologies rabbit poly-clonal, cat #3010), human anti-tubulin (Cell Signaling Technologies, rabbit poly-clonal, #2144), or SP1 (Cell Signaling Technologies, D4C3, cat #9389). Spectral Flow Cytemetry antibodies including vendor, clone, catalogue number, and dilution factor are listed in supplemental table 1. CITE-seq studies utilized TotalSeq-C cocktail resuspended and diluted per manufacture's instructions (Biolegend, catalogue number 399905). The following mouse antibodies were used for traditional flow cytometry studies (all at 1:200 dilution in PBS): anti-CD11c-eFluor506 (ThermoFisher, 69-0114-82, clone N418), anti-Ly6G-BrilliantViolet711 (Biolegend, 127643, clone 1A8), anti-CD11b-BrilliantViolet785 (Biolegend, 101243, clone M1/70), anti-Ly6C-PerCP/Cy5.5 (ThermoFisher, 45-5932-82, clone HK1.4), anti-CD103-PE/eFluor610 (ThermoFisher, 61-1031-82, clone 2E7) anti-NK1.1-PE/Cy7 (ThermoFisher, 25-5941-82, clone PK136), anti-CD11c-eFluor506 (ThermoFisher, 69-0114-82, clone N418) anti-CD11b-BrilliantViolet605 (Biolegend, 101257, clone M1/70), anti-CD8β-BrilliantViolet711 (Biolegend, 126633, clone YTS156.7.7) anti-MHCII-BrilliantViolet785 (Biolegend, 107645, clone M5/114.15.2), anti-GR-1-PerCP/Cy5.5 (ThermoFisher, 45-5931-80, clone RB6-8C5) anti-CD3-PE (Biolegend, 100206, clone 17A2),anti-NK1.1-PE/Cy7 (ThermoFisher, 25-5941-82, clone PK136) anti-CD4-AlexaFluor647 (Biolegend, 100530, clone RM4-5) anti-CD19-AlexaFluor700 (Biolegend, 115527, clone 6D5), anti-B220 BV510 (Biolegend, 103248, clone RA3-6B2), anti-CD45.2 FITC (Biolegend, 109805, clone 104), Anti-CD23 BV786 (BD Bioscience, 563988, clone B3B4), anti-CD93 PerCPCy5.5 (Biolegend, 136512, clone AA4.1), anti-Ly-6G biotin (Biolegend, 127603, clone 1A8), anti-CD11b biotin (Biolegend, 101204, cloneM1/70), anti-Ter119 biotin (Biolegend, 116204, clone TER-119), anti-CD3e biotin (Biolegend, 100304, clone 145-2C11), anti-B220 BV605 (Biolegend, 103244, clone RA3-6B2), anti-CD43 FITC (ThermoFischer, 11043185, clone eBioR2/60), anti-IgM PE (ThermoFischer, 12579082, clone II/41), anti-IgD BV711 (Biolegend, 405731, clone 11-26c.2a), Streptavidin eF450 (Invitrogen, 48431782), anti-BP-1 BV786 (BD Bioscience, 740882, clone BP-1), anti-CD24 BV711 (BD Bioscience, 563450, clone M1/69), anti-CD19 PE-Cy7 (Invitrogen, 25019382, clone eBio1D3 (1D3), anti-CD48 PerCP-Cy5.5 (Biolegend, 103422, clone HM48-1), anti-Sca-1 (Biolegend, 108114, clone D7), anti-c-Kit BV711 (Biolegend, 105835, clone 2B8), anti-CD150 PE (Biolegend, 115904, clone TC15-12F12.2), anti-CD16/32 BV421 (Biolegend, 101331, clone 93), anti-CD64 BV605 (Biolegend, 139323, clone X54-5/7.1), anti-CD32B PE (eBioscience, 12032182, clone AT130-2), anti-CD16.2 AF700 (Biolegend, 149507, clone 9E9).
Validation	For commercially available antibodies, validation has been performed by the manufacturer and corresponding certificates of analysis are available at the manufacturer's website (https://www.biolegend.com/; https://www.fortislife.com/bethyl-laboratories; https://www.cellsignal.com/; https://www.thermofisher.com/us/en/home.html; https://www.thermofisher.com/us/en/home/brands/invitrogen.html). Reactivity of primary antibodies used in flow cytometry assays was validated by the manufacturer in immunocytochemistry and frozen immunohistochemistry.

Eukaryotic cell lines

Policy information about cell lines and Sex and Gender in Research

Cell line source(s)	HEK293T were obtained from American Type Culture Collection (CRL-3216 ATCC; Manassas, VA) and cultured in Dulbecco's modified Eagle medium with 10% fetal bovine serum (FBS). HNT-34 (purchased from DSMZ, catalog no. ACC 600) and 5637 (purchased from ATCC, catalog no. HTB-9) cells were cultured in RPMI 1640 with 10% FBS. MUTZ-3 cells (purchased from DSMZ, ACC 295) were cultured in a minimum essential medium (with ribo- and deoxyribonucleosides)/20% FBS and 20% conditioned medium of cell line 5637. YCU-AML1 cells (gift from Dr. Hideaki Nakajima) were cultured with OP-9 (purchased from ATCC, CRL-2749) in Iscove's modified Dulbecco's medium with 10% FBS, 55 mM ⍰-mercaptoethanol (Sigma-Aldrich) and 20 ng/mL granulocyte-macrophage colony-stimulating factor (PeproTech). U937 wild-type (CRL-1593.2 ATCC) and U937 CD32A knockout cells generated previously44 and CD32A re-expression was achieved via introduction of the full-length sequence or a version with in-frame deletion of the transmembrane domain cloned into the PiggyBac vector. Murine RN2 cells (MLL-AF9 + NRASG12D) were generated as previously described45 and cultured in RPMI + 10% fetal bovine serum + 1% penicillin/streptomycin and passaged every 2-3 days to maintain a density of less than 1 x 106 cells per mL. No further authentications were performed. No commonly misidentified cell lines were used in the study.
Authentication	Cell lines were purchased directly through ATCC or DSMZ where certificates of authentication are provided. No further authentications were performed otherwise.
Mycoplasma contamination	All cell lines were negative for mycoplasma.
Commonly misidentified lines (See ICLAC register)	No commonly misidentified cell lines were used in the study.

Animals and other research organisms

Policy information about studies involving animals; ARRIVE guidelines recommended for reporting animal research, and Sex and Gender in Research

Laboratory animals	8-10 week-old CD45.1+ mice (Jackson Lab), inv(3)(3q21q26) mouse strain34 (RBRC09508; RIKEN BRC, National BioResource Project of the MEXT/AMED, Japan)
Wild animals	No wild-animals were used in this study.
Reporting on sex	Male and female mice were used as donors for bone marrow transplantation studies.
Field-collected samples	No field collected samples were used in this study.
Ethics oversight	All mouse procedures were completed in accordance with the Guidelines for the Care and Use of Laboratory Animals and were approved by the Institutional Animal Care and Use Committees at MSKCC. All mouse experiments were performed in accordance with a protocol approved by the MSKCC Institutional Animal Care and Use Committees (13-04-003).

Note that full information on the approval of the study protocol must also be provided in the manuscript.

Flow Cytometry

Plots

Confirm that:

☒ The axis labels state the marker and fluorochrome used (e.g. CD4-FITC).

☒ The axis scales are clearly visible. Include numbers along axes only for bottom left plot of group (a 'group' is an analysis of identical markers).

☒ All plots are contour plots with outliers or pseudocolor plots.

☒ A numerical value for number of cells or percentage (with statistics) is provided.

Methodology

Sample preparation	Surface-marker staining of murine hematopoietic cells was performed by first lysing cells with ACK lysis buffer and washing cells with ice-cold PBS. Cells were stained with antibodies in PBS for 25 minutes on ice. For hematopoietic stem/progenitor staining, cells were stained with a lineage cocktail cocktail comprised of antibodies targeting CD3, B220, Ter119, Gr-1, and CD11b and cells were also stained with c-Kit, Sca1, CD150, and CD48. For general immunophenotyping cells were stained with CD3, CD4, CD8b, CD11c, CD11b, MHC-II, Gr1, CD103, NK1.1, CD19, and U5 snRNP200 (after FcR block with CD16/32). For immunophenotyping with Fc receptor profiling, cells were stained with CD3, B220, CD11b, Ly6G, Ly6C, CD11c, NK1.1, MHC-II, CD16/32, CD64, CD32B, CD16.2, and U5 snRNP200. For B cell phenotyping cells were stained with cocktail 1 containing Gr-1, CD11b, Ter119, CD3e, B220, CD43, IgM, IgD, and snRNP200 or cocktail 2 containing Gr-1, CD11b, Ter119, CD3e, B220, CD43, BP-1, CD24, CD93, CD19, and U5 snRNP200.<br>Human bone marrow specimens were collected and processed in the Memorial Sloan Kettering Human Oncology Tissue Bank. Briefly, samples were subjected to Ficoll gradient for isolation of the mononuclear layer prior to being viably frozen and stored in liquid nitrogen. Sample thawing and preparation for spectral flow cytometry application described in detail in the methods section.
Instrument	Aurora (5 Laser configuration, Cytek Bioscience), Attune NxT Acoustic Focusing Cytometer (Lasers: BRV6Y, Thermo Fischer Scientific), BD FACS ARIA II (BD Bioscience)
Software	SpectroFlow Flow Cytometry Software (Cytek Bioscience), Attune NxT Flow Cytometry Software (Thermo Fischer Scientific), FACS Diva Software flow cytometry sorting (BD Biosciences), FlowJo (TreeStar)
Cell population abundance	For CRISPR Genome Wide Screen featured in figure 3d, GFP positive cells (containing the lentivirus vector used for library delivery) were sorted from the bulk population on day 2 after library introduction. Then after an 8 day culture period, GFP+ cells gated from the whole live cell population stained with anti-U5 snRNP200 APC antibody were sorted to allow for isolation of the highest top 10% U5 snRNP200+ cells and the lowest/negative bottom 10% U5 snRNP200 cells. Sorting and post sort analysis for purity of at least 99% performed by the Memorial Sloan Kettering Flow Cytometry Core.
Gating strategy	UMAP gating strategies illustrated and described in detail in supplemental figure 1. Gating strategies for quantification and analysis of human immune cell subsets per reference provided (Park. et. al. OMIP-69, Forty-Color Full Spectrum Flow Cytometry Panel for Deep Immunophenotyping of Major Cell Subsets in Human Peripheral Blood, Cytometry A . 2020 Oct;97(10):1044-1051. doi: 10.1002/cyto.a.24213.).<br>Gating strategies for analysis of murine immune cell subsets in and hematopoietic stem cell populations in supplemental figure 4 as previously described (Bournazos et. al., Fc-optimized antibodies elicit CD8 immunity to viral respiratory infection, Nature. 2020 Dec;588(7838):485-490. doi: 10.1038/s41586-020-2838-z. and Inoue et. al., Minor intron retention drives clonal hematopoietic disorders and diverse cancer predisposition. Nat Genet. 2021 May;53(5):707-718. doi: 10.1038/s41588-021-00828-9. Epub 2021 Apr 12.).<br>Gating strategies for analysis of murine immune cell subset Fc receptor expression illustrated and described in supplemental figure 5.

Gating strategies for murine B cell populations per reference provided (Harris et. al. ,Flow Cytometric Characterization of Murine B Cell Development, J. Vis. Exp. (167, e61565, doi:10.379/61565 (2021).).

☒ Tick this box to confirm that a figure exemplifying the gating strategy is provided in the Supplementary Information.
