# Supplementary figures and images for: Systematic evaluation of AML-associated antigens identifies anti-U5 SNRNP200 therapeutic antibodies for the treatment of acute myeloid leukemia
Source: Nat Cancer. 2023 Oct 23;4(12):1675–92. doi: 10.1038/s43018-023-00656-2 (PMC10733148; doi:10.1038/s43018-023-00656-2)

Fig. 4b

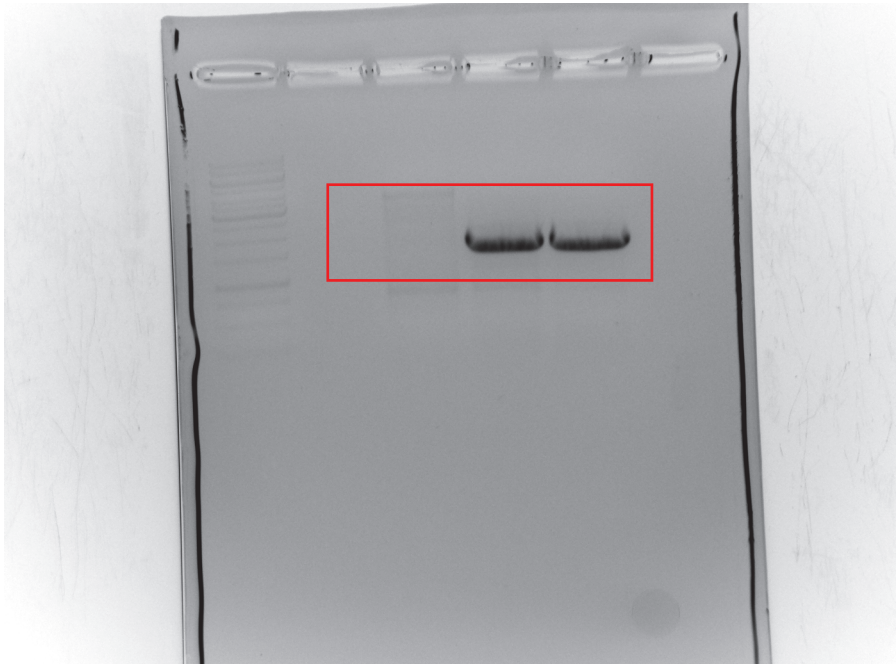

Halo tag

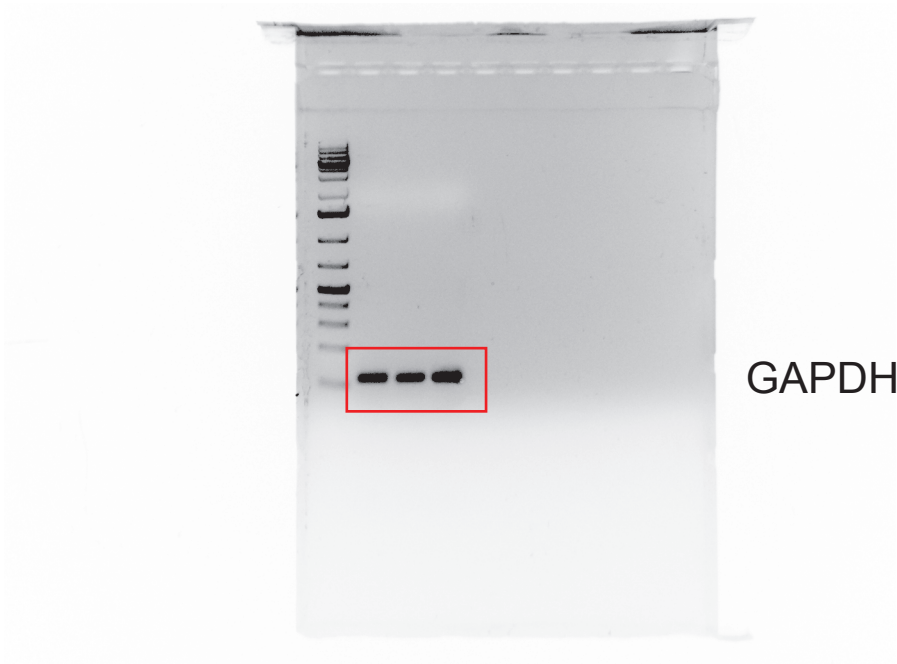

GAPDH

Fig. 4c

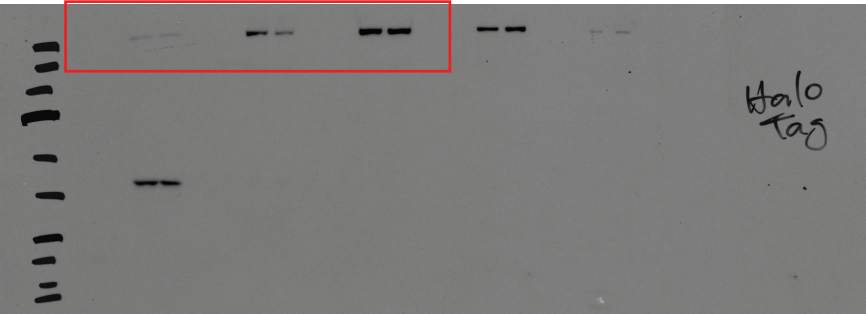

Halo tag

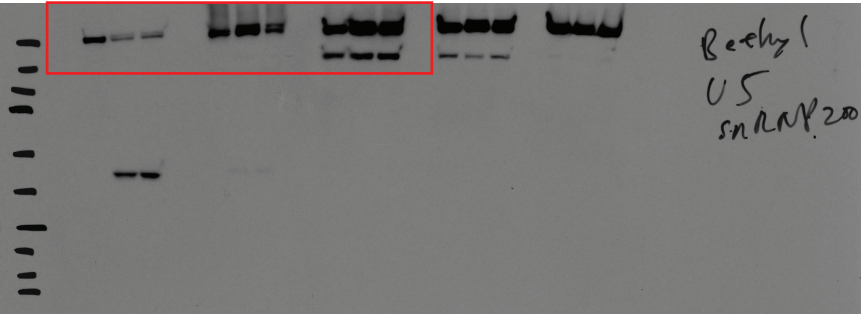

U5 snRNP200

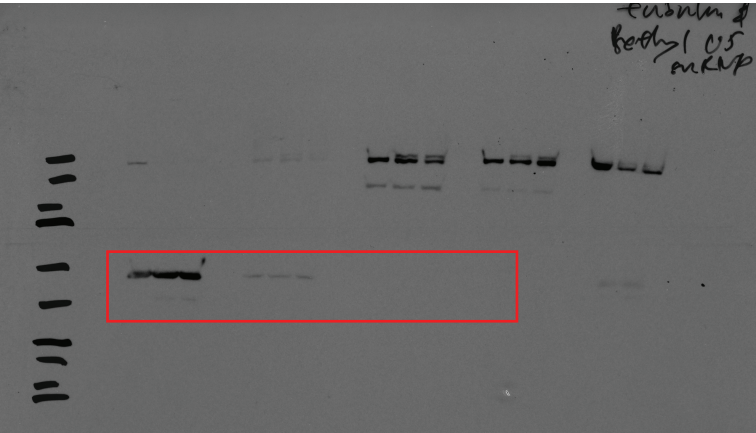

Tubulin

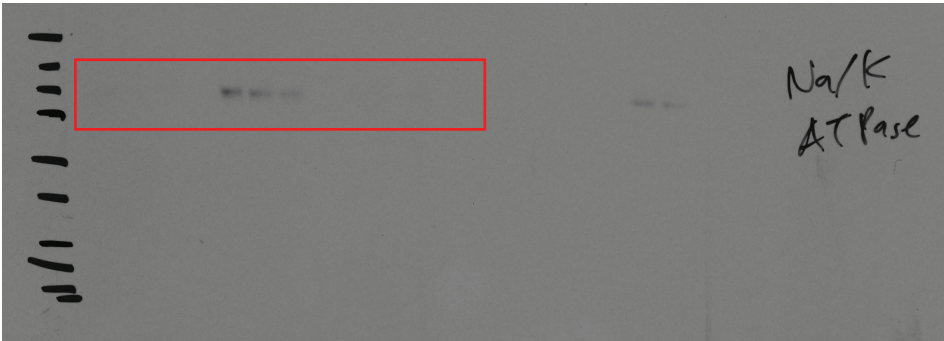

Na/K ATPase

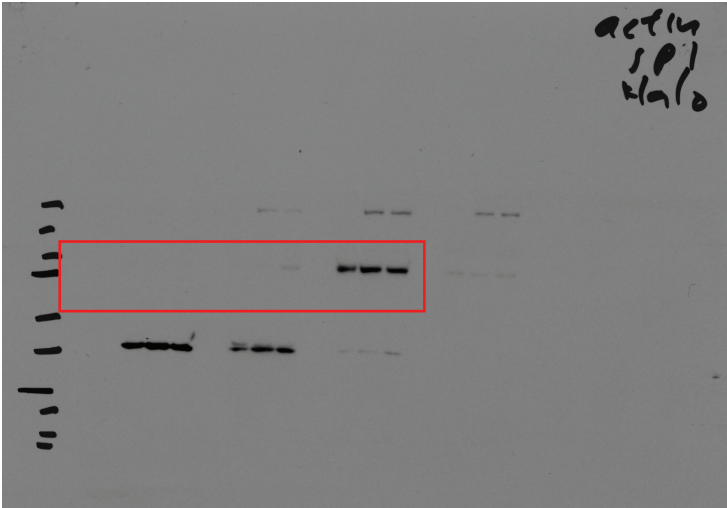

SP1

Supplement: Supplementary file 4 — Unprocessed western blots and gels. [file 43018_2023_656_MOESM4_ESM.pdf]

Fig. 6c

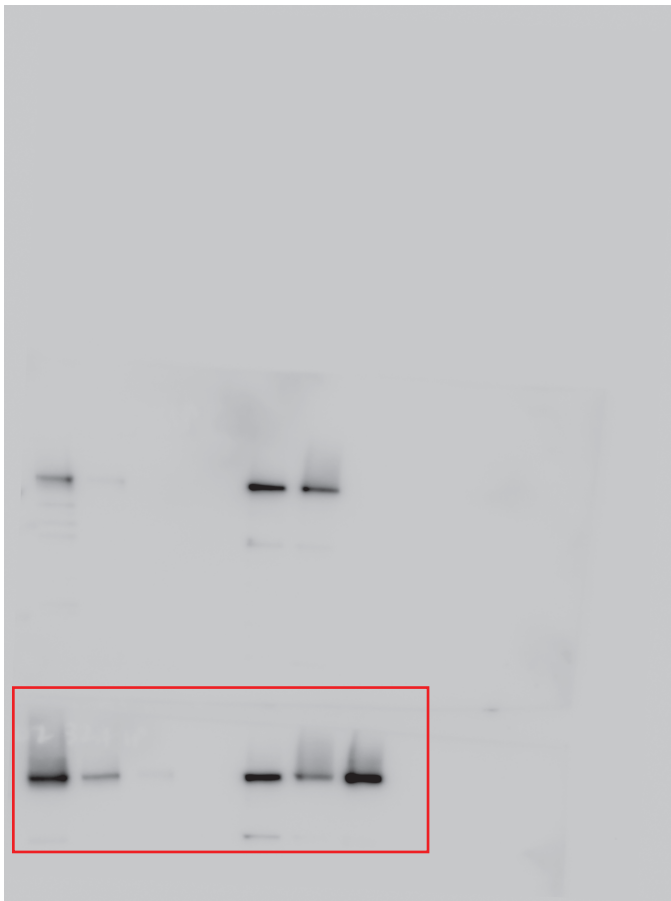

IP CD32A/WB U5 snRNP200

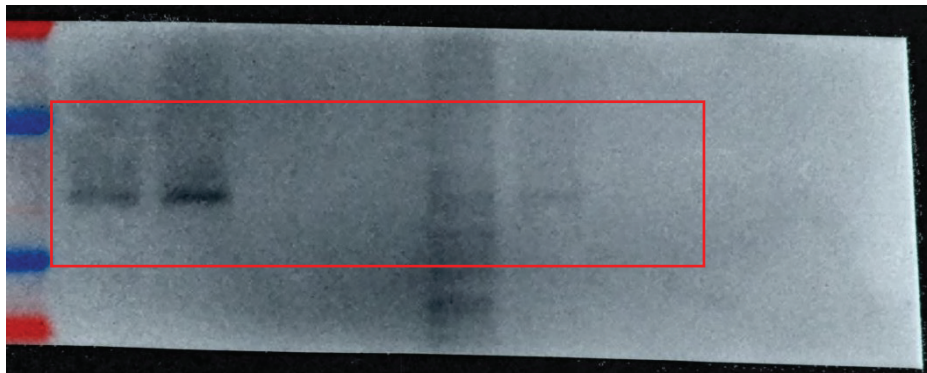

IP CD32A/WB CD32A

Supplement: Supplementary file 5 — Unprocessed western blots and gels. [file 43018_2023_656_MOESM5_ESM.pdf]
